# Evaluation of *Quercus infectoria* Phytoconstituents Against Oral Cancer: Network Pharmacology, Docking Simulation, and In Vitro Cytotoxicity Assay

**DOI:** 10.1002/fsn3.70857

**Published:** 2025-09-08

**Authors:** Priyanka Kamaria, Priyanka Tiwari, Prabitha Prabhakaran, Sakshi Bhardwaj, Krishna Kolachi, Shankar Thapa

**Affiliations:** ^1^ Department of Pharmaceutical Chemistry KLE College of Pharmacy, Bengaluru, KLE Academy of Higher Education and Research Belagavi Karnataka India; ^2^ Department of Pharmaceutical Chemistry JSS College of Pharmacy Mysuru Karnataka India; ^3^ Department of Life Science Altem Technologies Pvt. Ltd Bangalore Karnataka India; ^4^ Department of Pharmacy Universal College of Medical Sciences Bhairahawa Nepal

**Keywords:** cytotoxicity, DFT, molecular docking, network pharmacology, oral cancer, *Quercus Infectoria*

## Abstract

Oral cancer (OC) remains a significant global health concern due to its high incidence and mortality. *Quercus infectoria* (QI), a traditional medicinal plant, harbors bioactive compounds with potential anticancer properties. This study systematically integrates multiple computational and experimental approaches to identify and validate the anticancer potential of QI phytoconstituents. Network pharmacology was used to predict key molecular targets and signaling pathways, notably apoptosis and the PI3K‐Akt pathway—providing mechanistic validation through pathway‐level insights into anticancer activity. Molecular docking revealed strong binding affinities of Nyctanthic acid (−125.426 and −113.841 kcal/mol) and β‐Glucogallin (−96.7558 and −112.534 kcal/mol) with AKT1 and GAPDH, respectively. Molecular dynamics (MD) simulations further confirmed the structural validation by demonstrating the stability and conformational integrity of these protein‐ligand complexes. Cellular validation was achieved through cytotoxic assay on KB oral cancer cells, which showed dose‐dependent cytotoxicity, with IC_50_ values of 224.41 ± 2.01 μg/mL for the QI extract and 213.77 ± 1.98 μg/mL for Cisplatin. Density Functional Theory (DFT) analysis further supported the electronic stability and reactivity of Nyctanthic acid. From the study, Nyctanthic acid showed the superior binding affinity, target relevance (AKT1), and computational stability that make it a promising multi‐target anticancer candidate. These findings lay the groundwork for future in vivo studies, formulation development, and translational drug development.

## Graphical abstract

This study illustrates a workflow exploring *Quercus infectoria* against oral cancer through network pharmacology, target prediction, molecular docking, DFT, molecular dynamics, and pathway enrichment. Molecular docking showed strong binding affinities of Nyctanthic acid and β‐Glucogallin with AKT1 and GAPDH, respectively, with the Nyctanthic acid‐AKT1 interaction showing the highest binding energy (−125.426 kcal/mol). Furthermore, MTT assays on KB oral cancer cells revealed dose‐dependent cytotoxicity, with IC_50_ values of 224.41 ± 2.01 μg/mL for QI extract and 213.77 ± 1.98 μg/mL for Cisplatin.

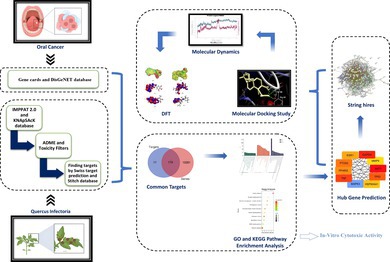

**Abbreviations**
ADME: absorption, distribution, metabolism, and excretion
AKT1: serine/threonine protein kinase 1
BP: biological process
CC: cellular component
DFT: density functional theory
GAPDH: glyceraldehyde‐3‐phosphate dehydrogenase
GO: Gene Ontology
IMPPAT‐2.0: Indian Medicinal Plants, Phytochemistry and Therapeutics‐2.0
KEGG: Enrichment and Kyoto Encyclopedia of Genes and Genomes
MD: molecular dynamics
MF: molecular function
MolSA: molecular surface area
OC: oral cancer
OSCC: oral squamous cell carcinoma
PPI: protein–protein interaction
PSA: polar surface area
QI:
*Quercus infectoria*

QMM: quantum mechanical methods
rGyr: radius of gyration
RMSD: root mean square deviation
RMSF: root mean square fluctuation
ROS: reactive oxygen species
SASA: solvent‐accessible surface area
VEGF: vascular endothelial growth factor

## 1 Introduction

Due to high mortality and morbidity rates, oral cancer (OC) represents a significant health burden worldwide. It can affect people of any racial, ethnic, or geographical background. It is well known that this kind of cancer progresses rapidly and undergoes frequent changes in appearance. Additionally, it may develop the capacity to disseminate to other bodily parts, including lymph nodes and other organs or tissues (Jayaraman et al. 2023). Oral cancer, which includes malignancies of the lip, oral cavity, and oropharynx, ranks as the 13th most common cancer globally. In 2022, cancers of the lip and oral cavity accounted for an estimated 188,438 deaths worldwide. Trends indicate a potential increase in incidence, particularly in low and lower‐middle‐income countries, by 2050. Notably, approximately 52% of these deaths occurred in South and South‐East Asia, highlighting a significant regional burden (Walter 2022; Warnakulasuriya and Filho 2025). The American Cancer Society estimates 59,660 new cases and 12,770 deaths from oral cavity and oropharyngeal cancers in the United States for 2025 (Oral Cavity and Oropharyngeal Cancer Key Statistics 2021; American Cancer Society 2025). While oral cancer constitutes 1%–2% of all cancer cases in high‐income countries like the UK, USA, and Japan (Seethalakshmi 2013), it presents a far greater public health concern in India (Akashanand et al. 2024). The development of oral cancer has been linked to both genetic and epigenetic alterations, often triggered by modifiable risk factors such as tobacco use (smoking or chewing), excessive alcohol consumption, and poor oral hygiene. Socioeconomic disparities further influence disease outcomes; for instance, studies indicate that Medicaid beneficiaries in the United States experience higher incidence, prevalence (Wang et al. 2019), and mortality related to oral and oropharyngeal cancers compared to those with commercial insurance (Lingen et al. 2008; Tranby et al. 2022). This indicates a need for effective anticancer agents.

Synthetic anticancer agents are often associated with adverse effects (Basak et al. 2022). Therefore, natural products derived from diverse sources and geographical regions can be the better choice for study. Natural products have long been utilized in the treatment of numerous debilitating human diseases (Naeem et al. 2022). Traditional medicinal plants have long served as valuable resources in drug discovery due to their diverse bioactive compounds and therapeutic potential. Among the modern tools that facilitate the scientific validation of such plant‐based remedies are computational approaches like molecular docking, network pharmacology, molecular dynamics simulation, and quantum mechanical modeling. These methods have significantly advanced our understanding of the pharmacological mechanisms underlying traditional medicine, especially in Unani Ayurveda and Chinese medicine systems (Elham et al. 2021; Lukman et al. 2007; Ruchawapol et al. 2022; Thapa et al. 2025; Xiaoying et al. 2023). The use of natural products in cancer treatment has proven to be highly effective. For instance, paclitaxel (Taxol), originally derived from the bark of the Pacific yew tree ( *Taxus brevifolia* ), has become a widely used chemotherapy agent for treating ovarian, breast, and lung cancers (Kaveh Zenjanab et al. 2024; Weaver 2014). Another success story is vincristine, which is derived from the Madagascar periwinkle ( *Catharanthus roseus* ) and has been used for decades to treat childhood leukemia and other cancers (Awosika et al. 2023). More recently, camptothecin and its derivatives, such as topotecan, have shown promising anticancer activity, particularly for ovarian and small‐cell lung cancers (Movahed et al. 2021).

Among the diverse natural products, *Quercus infectoria* (QI) exhibits anti‐inflammatory, hypoglycemic, hypocholesterolemic, antihypertensive, antimicrobial, antiproliferative, and antioxidant activities (Banc et al. 2023; Wan Nor Amilah et al. 2022). Quercus is a forest tree species with 600 species worldwide. It is a member of the Fagaceae family. They are dispersed throughout the various northern hemisphere regions (Taib et al. 2020). The species of Quercus plants in this genus determine their morphology. Certain Quercus species grow to be enormous trees, while others are little trees or shrubs. The galls have historically been used in Malaysian traditional medicine to stimulate vaginal contractions and restore postpartum uterine elasticity (Mahboubi 2020). Traditionally, it has been used in India for the treatment of gum disease and various oral cavity disorders. In addition to its therapeutic applications, it also offers several health benefits, such as anti‐inflammatory and antioxidant properties, which contribute to overall oral and systemic health (Ansari Shaiqua et al. 2016; Jain et al. 2019).

Network pharmacology is an emerging interdisciplinary field that combines systems biology, bioinformatics, and pharmacology to provide a comprehensive approach to drug discovery. Unlike the traditional one‐drug‐one‐target model, it focuses on the complex interactions between drugs, genes, proteins, and disease pathways (Zhang et al. 2019). By analyzing these intricate molecular networks, network pharmacology enables the identification of multiple targets and pathways associated with a single compound, offering a more comprehensive understanding of drug mechanisms and disease pathogenesis (Zhang et al. 2020). Molecular dynamics represents a method of simulating physical motions of atoms and molecules in a computer over a predetermined amount of time, which sheds light on the dynamic evolution of the system (Kushwaha et al. 2021). One of the best and most effective Quantum Mechanical Methods (QMM) for the drug design process is Density Functional Theory (DFT) (Bellahcene et al. 2024). When compared to other computational methods, this one requires less time and money. DFT is quite significant in the drug design field. Receptor‐drug interaction modeling is one of the few examples demonstrating the function and significance of DFT, modeling of organometallic drug, and modeling of the mechanism of action of the drug. DFT‐based calculations are emerging as a key methodological replacement in theoretical medicinal chemistry. The fundamental tenet of DFT is that the ground state energy and other molecular properties are solely determined by the electronic density, which serves as a representation of the density functional, mapping a function to a value (Tandon et al. 2019). The electronic properties of a molecule, such as its electron density distribution, molecular orbitals, and frontier molecular orbitals (HOMO and LUMO), are crucial in determining how a compound interacts with biological targets, such as enzymes or receptors involved in cancer progression (Kirishnamaline et al. 2021). These properties influence factors like the molecule's ability to bind to DNA, proteins, or other cellular structures, as well as its potential for forming reactive intermediates that can induce cell death (cytotoxicity). Additionally, understanding the reactivity of a molecule is essential for predicting its metabolic stability and its ability to evade detoxification mechanisms in the body (Mu and Gao 2022). In terms of drug‐likeness, DFT calculations can help predict the stability and reactivity of molecules under physiological conditions, which is critical for designing drugs with optimal pharmacokinetic properties (e.g., bioavailability, solubility, and permeability) (Afridi et al. 2024). DFT allows evaluating how structural changes might improve or hinder these properties, leading to more effective and safer drug candidates (El‐Shamy et al. 2022).

In this context, QI has attracted growing scientific interest due to its traditional (Unani system of medicine) use and rich phytochemical profile. The galls of QI are particularly known to contain a variety of polyphenolic compounds, including gallic acid, ellagic acid, and tannins, which have demonstrated potent antioxidant, antimicrobial, and anti‐inflammatory properties (Banc et al. 2023). More importantly, recent studies suggest that these compounds also exhibit anticancer effects, particularly by modulating cell cycle progression, inducing apoptosis, and inhibiting angiogenesis (Hadidi et al. 2024; He et al. 2021). An in vitro study by Ismail et al. reported the inhibition of the HeLa cell line from the QI extract (Ismail et al. 2021). Furthermore, gallic acid has been reported to inhibit cancer cell proliferation by activating caspase pathways and suppressing PI3K/Akt signaling in oral squamous cell carcinoma cells (Ko et al. 2022). Similarly, ellagic acid has been shown to inhibit metastasis in a multitarget mechanism in cancer models (Lu et al. 2023). Among the numerous compounds reported in QI, Nyctanthic acid and β‐Glucogallin have recently garnered scientific interest due to their structural characteristics and reported biological activities. Nyctanthic acid, a triterpenoid acid, aligns with a class of compounds known to induce apoptosis, cause cell cycle arrest, and inhibit NF‐κB signaling in diverse cancer types (Petronelli et al. 2009; Zhang et al. 2014). β‐Glucogallin, a galloyl‐glucose derivative, functions as a potent antioxidant, anti‐inflammatory agent, and aldose reductase inhibitor with emerging evidence of anti‐proliferative activity against cancer cell lines (Puppala et al. 2012; Singh et al. 2022). Despite these promising reports, the molecular mechanisms and multitargeted actions of QI's phytochemicals in the context of oral cancer remain insufficiently explored. Nyctanthic acid and β‐Glucogallin have some reported biological activities; their specific roles in oral cancer, particularly from *Quercus infectoria* , remain underexplored.

Therefore, in this comprehensive in silico–in vitro study, we systematically investigate selected bioactive compounds from QI through molecular docking, network pharmacology, molecular dynamics simulations, and DFT analysis. This comprehensive approach aims to elucidate the interaction of QI constituents with key oral cancer targets, assess their pharmacokinetic suitability, and explore their dynamic behavior and electronic properties.

## 2 Material and Methods

### 2.1 Plant Material and Authentication

The dried galls of *Quercus infectoria* were procured from a certified Ayurvedic raw material supplier, Natural Remedies Pvt. Ltd., Bangalore, India, which complies with AYUSH and GMP quality standards. The plant material was selected based on morphological characteristics such as size, color, surface texture, and hardness, in accordance with the Ayurvedic Pharmacopeia of India (Vol. II, Part I). Authentication of the plant material was carried out by Dr. S. Ramesh, Taxonomist, Department of Botany, Bangalore University, Bangalore, India. The authentication process involved macroscopic and microscopic examination of the galls, followed by comparison with standard pharmacognostic parameters. A voucher specimen (Specimen No: BU‐Herbarium‐2025‐QI‐07) has been deposited in the Herbarium of the Department of Botany, Bangalore University, for future reference.

### 2.2 Extraction of Galls of *Quercus infectoria*

The extraction process of *Quercus infectoria galls* was conducted using a cold maceration method to prepare the methanolic extract. To prepare the ethanolic extract, 100 g of finely powdered galls were soaked in 500 mL of methanol (Analytical grade) and left undisturbed at room temperature (approximately 25°C) for 24 h. Following the initial extraction, the mixture was filtered using (Whatman No. 1 filter paper), and the remaining solid residue was subjected to a second round of extraction using fresh ethanol. The filtrates from both extractions were pooled together. The combined filtrates were concentrated using a rotary evaporator at a temperature of 45°C and reduced pressure. The resulting semi‐solid extract was then dried in a hot air oven at 40°C until a powdered form was achieved. The final crude ethanolic extract was collected, weighed, and stored for further analysis (Fan et al. 2014).

### 2.3 Primary Screening of Phytoconstituents

Primary phytochemical screening of the *Q. infectoria* ethanolic extract was performed using standard qualitative methods to detect the presence of alkaloids, flavonoids, phenols, tannins, saponins, terpenoids, steroids, glycosides, and carbohydrates (Pandey, Thapa, Kaundinnyayana, and Panta 2024).

### 2.4 Network Pharmacology

#### 2.4.1 Screening QI and OC Related Targets

The active constituents of QI were retrieved from Indian Medicinal Plants, Phytochemistry And Therapeutics‐2.0 database (IMPPAT‐2.0) (https://cb.imsc.res.in/imppat/) and KNApSAcK: A Comprehensive Species‐Metabolite Relationship Database. Then these compounds were screened for drug likeness through the Molsoft tool (https://www.molsoft.com/) and SwissADME server (http://www.swissadme.ch/). To evaluate the drug‐likeness and pharmacokinetic viability of the selected bioactive compounds from *Quercus infectoria* , an in silico Absorption, Distribution, Metabolism, and Excretion (ADME) profiling was performed using platforms such as SwissADME (http://www.swissadme.ch/) and pkCSM (https://biosig.lab.uq.edu.au/pkcsm/). Specific parameters analyzed under absorption included human intestinal absorption (HIA), Caco‐2 cell permeability, and P‐glycoprotein (P‐gp) substrate or inhibition potential. Distribution‐related parameters assessed were blood‐brain barrier (BBB) permeability. For metabolism, the interaction of the compounds with key cytochrome P450 enzymes (CYP1A2 and CYP2C19) was evaluated to identify potential inhibitory effects. Toxicological properties were assessed by predicting hepatotoxicity and other organ toxicities. Drug‐likeness was further determined based on Lipinski's Rule of Five to predict oral bioavailability. Physicochemical characteristics such as molecular weight, lipophilicity (logP), topological polar surface area (TPSA), number of hydrogen bond donors and acceptors, and the number of rotatable bonds were also evaluated to estimate membrane permeability and solubility (Supporting Information, Table S1). Although quantitativestructure‐activity relationship (QSAR) analysis was considered, it was not implemented in this study due to the lack of experimentally validated anticancer activity data specific to Nyctanthic acid and β‐Glucogallin in oral cancer models. The screening limits were kept for drug likeness model score ≥ 0.18 and bioavailability score ≥ 0.3, respectively (Gfeller et al. 2013). The compounds were screened for toxicity prediction through ProTox 3.0 (https://tox.charite.de/protox3/)–prediction of toxicity of chemicals. Finally, compounds screened through the above process (2 compounds) were subjected to target prediction through SwissTargetPrediction (http://www.swisstargetprediction.ch/) and STITCH database. Then genes for oral cancer were collected by searching through gene cards and DisGeNET database (https://disgenet.com/). Then it was followed by taking the intersecting gene and target set between SwissTarget and DisGeNET.

#### 2.4.2 Protein–Protein Network Construction

The common targets between QI and OC were observed by drawing a Venn diagram through Venny v2.1 (https://bioinfogp.cnb.csic.es/tools/venny/). The common genes were used to construct a PPI through the STRING v12.0 database (https://string‐db.org/). In the PPI network construction process, each gene was represented as a node. Edges between nodes were defined based on known and predicted interactions from the STRING database, where interactions can be either experimentally determined or predicted through computational methods. We selected experimental interactions. These interactions were visualized in the network, where the strength of each interaction is determined by a confidence score (ranging from 0 to 1, with 1 being the highest confidence level). For the pathway enrichment analysis, the statistical parameters were set as follows: a *p*‐value cutoff of 0.05 was used to determine statistical significance. To control for multiple testing, the False Discovery Rate (FDR) correction was applied, ensuring that the results account for the number of tests conducted.

#### 2.4.3 Gene Ontology (GO) Enrichment and Kyoto Encyclopedia of Genes and Genomes (KEGG)

The GO and KEGG enrichment analysis were done through DAVID v2024 database (https://davidbioinformatics.nih.gov/) for finding out biological pathways, cellular component and molecular function term involved. The protein–protein interaction (PPI) network for target genes was constructed by the STRING database, and further hub genes were identified by the Cytohubba module of Cytoscape v3.10.0 (Chen et al. 2022). The top 10 genes ranked by degree were selected for further network construction with the help of Cytoscape v3.10.0.

### 2.5 Molecular Docking Study

#### 2.5.1 Ligand Selection and Preparation

The selection of phytoconstituents from QI was conducted through a comprehensive literature review and cross‐referencing with reputable natural compound databases such as PubChem and IMPPAT 2.0. An initial pool of 29 phytochemicals reported in QI was compiled based on their documented presence in galls and potential pharmacological relevance (Vivek‐Ananth et al. 2023). The selection of phytoconstituents from QI was guided by their pharmacokinetic and structural properties, as assessed using NP‐likeness score, bioavailability score, Lipinski's Rule of Five, and shape complexity. Among the screened compounds, Nyctanthic acid and β‐Glucogallin were prioritized for molecular docking and further in silico analysis as Nyctanthic acid exhibited the highest NP‐likeness score (3.171), a high oral bioavailability score (0.85), and passed Lipinski's Rule of Five, indicating excellent drug‐likeness and natural scaffold (Table 1). It also demonstrated significant shape complexity (0.83), supporting specific target binding. Notably, Nyctanthic acid has also been reported to exert cytotoxic and anti‐proliferative activity against cancer cell lines (Parekh and Soni 2020). β‐Glucogallin, although having a moderate bioavailability score (0.11), showed a high NP‐likeness score (1.925) and complied with Lipinski's criteria, suggesting a favorable balance between structural features and potential biological activity. Most importantly, the selection was supported by literature describing its antioxidant, anti‐inflammatory, and anticancer potential through modulation of oxidative stress pathways (Khan, Singh, Bhattacharya, Chakravarti, et al. 2022). Moreover, β‐Glucogallin is structurally related to gallic acid derivatives known to interfere with cancer‐related signaling cascades (Khan, Singh, Bhattacharya, Kumar, et al. 2022; Sarı et al. 2024). These parameters, along with literature‐reported pharmacological relevance, formed the basis for their selection in docking and molecular dynamics simulations.

**TABLE 1:** IMPPAT 2.0 parameters to select Nyctanthic acid and β‐Glucogallin for docking study.

SN	Phytoconstituents	NP‐Likeness score	Bioavailability score	Lipinski's rule of 5	Shape complexity
1	Syringic acid	0.544	0.56	Pass	0.22
2	Flavylium	0.574	0.55	Pass	0
3	β‐Glucogallic acid	1.925	0.11	Pass	0.46
4	Ellagic acid	1.071	0.55	Pass	0
5	Amentoflavone	1.234	0.17	Failed	0
6	Nyctanthic acid	3.171	0.85	Pass	0.83
7	Methyl oleanolate	3.175	0.55	Pass	0.9
8	Tannic acid	0.379	—	Failed	0.08
9	Gallic acid	0.981	0.56	Pass	0
10	Methyl betulate	2.993	0.55	Pass	0.9
11	β‐Sitosterol	2.681	0.55	Pass	0.93

The standard reference compound Galuteolin, known for its anticancer properties, was included for comparative analysis. The 2D structures of all compounds were retrieved from PubChem (https://pubchem.ncbi.nlm.nih.gov/) in SDF format and converted into 3D PDB format using Open Babel. The ligands were then energy‐minimized using MMFF94 force field to remove steric hindrances and optimize the geometry. The prepared ligands were saved in PDBQT format for docking studies (Kurmi et al. 2025).

#### 2.5.2 Protein Selection and Preparation

The hub gene analysis from the protein–protein interaction (PPI) network identified AKT1 (AKT serine/threonine kinase 1) and GAPDH (glyceraldehyde‐3‐phosphate dehydrogenase) as the most central and functionally relevant targets associated with oral cancer pathogenesis. AKT1 plays a key role in the PI3K/Akt signaling pathway, frequently implicated in cancer cell survival and proliferation. GAPDH, traditionally known as a glycolytic enzyme, also regulates apoptosis and is overexpressed in various tumors. The 3D structures corresponding to these targets were retrieved from the Protein Data Bank (https://www.rcsb.org/):

AKT1‐related protein: PDB ID: 4GV1 (Resolution: 1.49 Å) (Addie et al. 2013).

GAPDH‐related protein: PDB ID: 1U8F (Resolution: 1.75 Å) (Jenkins and Tanner 2006).

Protein structures were selected based on:

- High resolution (< 2.0 Å) to ensure structural accuracy.
- Completeness of polypeptide chains without missing regions.
- Presence of co‐crystallized ligands to guide binding site identification.

Protein preparation was performed using Molegro Virtual Docker (MVD 2013, version 6.0). The following steps were undertaken (Sağır et al. 2025):

- Water molecules and heteroatoms (e.g., co‐crystallized ligand, solvent molecules) were removed to prevent steric clashes and false‐positive interactions during docking, as these components may not accurately present physiological relevance unless directly involved in binding.
- Polar hydrogens were added to account for missing atoms often excluded in crystal structures (Gote et al. 2025).

Energy minimization was carried out using the internal force field of MVD to relax the structure and relieve potential steric strains introduced during processing. The active site was defined based on the binding pocket of co‐crystallized ligands and literature‐reported catalytic or regulatory residues.

#### 2.5.3 Protein Structure Validation

Protein structures were validated for quality using Ramachandran plot analysis via SAVES v6.1 (supports PROCHECK analysis) to ensure that the majority of residues were in the allowed regions, ensuring reliability of the structure for docking purposes. The 3D structure of both the proteins was validated through VERIFY3D (https://saves.mbi.ucla.edu/) (Pandey, Thapa, Biradar, et al. 2024).

#### 2.5.4 Docking Procedure

Molecular docking was performed using Molegro Virtual Docker (MVD) version 6.0 to evaluate the binding affinity and interaction profile of ligands with the active sites of target proteins. MVD version 6.0 employs the MolDock scoring function based on a hybrid search algorithm called guided differential evolution. This algorithm efficiently explores the conformational space of ligands within the active site of the receptor. The binding affinity is an energy‐based scoring function derived from the Piecewise Linear Potential (PLP), which estimates the binding affinity by considering van der Waals forces, hydrogen bonding, electrostatic interactions, and internal ligand energy (Thomsen and Christensen 2006). The docking parameters were set to a population size of 50, maximum iterations of 1500, and a grid resolution of 0.30 Å. Each ligand was docked individually into the predefined active site (blind docking) of both proteins (Xia et al. 2021). The best poses were selected based on the binding affinity, which reflects the binding affinity, and further evaluated for hydrogen bonding, hydrophobic, and electrostatic interactions using 2D and 3D visualization tools PyMOL v3.1 (Unsal, Oner, et al. 2025). The results were compared with the binding behavior of the standard drug Galuteolin and Cisplatin. Galuteolin is a natural product and suppresses cell proliferation (Guan et al. 2020). No negative control was used in docking.

### 2.6 Validation of Docking Protocol

To ensure the reliability and accuracy of our molecular docking protocol, a native ligand re‐docking procedure was conducted. Specifically, the co‐crystallized ligands from the crystal structures of AKT1 (PDB ID: 4GV1) and GAPDH (PDB ID: 1U8F) were extracted and then re‐docked into their respective active sites using the same docking parameters employed for the selected phytoconstituents. The resulting docked pose was then superimposed onto the original crystal structure to evaluate structural accuracy. The root mean square deviation (RMSD) between the re‐docked and crystallographic ligand poses was found to be < 2.0 Å in both cases (for AKT1 = 1.79 and for GAPDH = 1.60 Å), which is considered indicative of a valid docking procedure. This step ensured that the scoring function and grid parameters used were capable of accurately predicting ligand binding orientations. Additionally, all bioactive compounds screened in the study were docked using the validated protocol, and binding affinities were compared alongside key interactions (e.g., hydrogen bonds, hydrophobic contacts) within the protein active site (Gote et al. 2025).

### 2.7 MD Simulation

The MD simulation provides insights into the natural dynamics of biomolecules in solution on various timescales. It can also determine the conformations of a molecule or a complex that are thermally accessible. MD simulations were performed for 100 ns using Desmond (Schrödinger 2020‐4). The initial stages of the protein and ligand complexes for MD simulations were obtained from the docking output files. The protein‐ligand systems were further prepared for simulation using the System Builder tool (Schrödinger 2022.1). A solvent model with an orthorhombic box and transferable intermolecular interaction potential 3 points (TIP3P) was selected. The OPLS2005 force field was used as a simulator. The models were neutralized by adding counter ions where needed. To mimic the physiological conditions, 0.15 M salt (NaCl) was added (Thapa et al. 2025). The NPT ensemble with 300 K temperature and one atm pressure was selected to complete the simulation process. The Desmond trajectories were saved after every 100 ps for analysis, and the stability of simulations was evaluated by calculating the RMSD, root mean square fluctuation (RMSF), and protein‐ligand contacts over time (Hansson et al. 2002). The stability of the protein‐ligand complex was also assessed by the overall deviation between the two which should be ideally < 2 Å (Thapa et al. 2023).

### 2.8 DFT Calculations

The Dipole, atomic charges, HOMO and LUMO energies, total energy, and other properties of molecules were found and optimized by using the density functional quantum mechanics method in DMol3. DMol3 is a Material Studio suite from BIOVIA Discovery Studio 2021 (Delley 2000; Quayum et al. 2024).

The Perdew–Burke–Ernzerhof (PBE) functional within the Generalized Gradient Approximation (GGA) was employed for the exchange‐correlation energy. The Double Numerical plus Polarization (DNP) basis set was used, which is comparable in accuracy to the 6‐31G** basis set in Gaussian‐type orbitals, but optimized for numerical calculations. All‐electron calculations were performed without any frozen‐core approximation. The following convergence criteria were applied during geometry optimization:

- Energy tolerance: 1 × 10^−5^ Ha
- Maximum force: 0.002 Ha/Å
- Maximum displacement: 0.005 Å

The self‐consistent field (SCF) cycle was considered converged when the change in total energy between successive iterations was < 1 × 10^−6^ Ha. A fine integration grid and global orbital cutoff radius of 5.0 Å were used to ensure computational accuracy (Tighadouini et al. 2018). This computational setup provides a robust and reliable framework for evaluating the electronic structure and reactivity descriptors of the studied molecules.

### 2.9 In Vitro Validation of Cytotoxicity Activity

The in vitro anticancer potential of QI extract was evaluated using the MTT (3‐[4,5‐dimethylthiazol‐2‐yl]‐2,5 diphenyl tetrazolium bromide) assay on the human oral cancer cell line KB, which is commonly used in cytotoxicity screening. The KB cell line (ATCC CRL‐3596) was procured from a certified cell repository, National Centre for Cell Sciences (NCCS) Pune, India, and the cells were cultured in Dulbecco's Modified Eagle Medium (DMEM) supplemented with 10% fetal bovine serum (FBS) and Phosphate Buffered Saline (PBS). Cells were maintained at 37°C in a humidified incubator with 5% CO_2_. For the assay, 50 μL of a 1 × 10^5^ cells/mL suspension was seeded into 96‐well plates and incubated for 24 h to allow adherence (Riss et al. 2016). QI extract and Cisplatin (positive control) were prepared in DMEM and added at concentrations ranging from 31.25 to 500 μg/mL in a 100 μL volume. A group of untreated cells, receiving only culture medium without any test compound, served as the negative control to represent 100% cell viability. Following 24‐h exposure, 20 μL of MTT reagent (5 mg/mL in PBS, pH 7.4) was added to each well and incubated in the dark for 4 h. After incubation, the supernatant was carefully removed, and the resulting formazan crystals were dissolved in 100 μL DMSO (Narayan Biswal et al. 2017). Absorbance was measured at 492 nm using a microplate reader. All treatments were performed in triplicate, and wells with untreated cells served as negative controls. The percentage of cell viability was calculated relative to control wells, and IC_50_ values were determined via nonlinear regression analysis (Biradar et al. 2023).

### 2.10 Statistical Analysis

Cell viability data were expressed as mean ± standard deviation (SD) from three independent experiments. To evaluate the significance of differences in % cell viability between the QI extract and the standard drug cisplatin at each concentration, an unpaired two‐tailed Student's *t*‐test was performed. The *t*‐test was used to determine whether the means of two independent groups were statistically different, under the assumption of normal data distribution and equal variances. A *p*‐value < 0.05 was considered statistically significant. The statistical analysis was performed in Microsoft Excel Office‐2019.

## 3 Results

### 3.1 Pharmacokinetic and Toxicity Screening

Nyctanthic Acid exhibited a high bioavailability (BA = 0.85) and drug‐likeness score (DS = 0.81), along with a high lipophilicity (Log *p* = 8.43), indicating strong membrane permeability. In contrast, β‐Glucogallin showed moderate bioavailability (0.55), lower drug‐likeness (0.52), and was hydrophilic in nature (Log *p* = −2.24). Neither compound was predicted to inhibit CYP1A2 or CYP2C19 enzymes, nor act as a P‐glycoprotein substrate, suggesting a low potential for metabolic interference or efflux‐related drug resistance (Table 2).

**TABLE 2:** Bioavailability score (BA), drug likeness score (DS), and pharmacokinetic properties of selected 2 compounds.

Active ingredients	PubChem ID	BA	DS	Log P	CYP1A2 inhibitor	CYP2C19 inhibitor	P‐gp substrate
Nyctanthic Acid	273516298	0.85	0.81	8.43	No	No	No
β‐Glucogallin	124021	0.55	0.52	−2.24	No	No	No

The toxicity assessment of Nyctanthic Acid and β‐Glucogallin indicated a favorable safety profile for both compounds. Neither ingredient showed evidence of carcinogenicity, immunogenicity, mutagenicity, or cytotoxicity. Additionally, both were classified as inactive in terms of hepatotoxicity, suggesting a low risk of liver damage (Table 3). These results support their potential suitability for therapeutic applications with minimal toxicological concerns.

**TABLE 3:** Predicted toxicity results of Nyctanthic acid and β‐Glucogallin.

SN	Toxicity	Phytoconstituents	
		Nyctanthic Acid	β‐Glucogallin
1	Carcinogenicity	No	No
2	Immunogenicity	No	No
3	Mutagenicity	No	No
4	Cytotoxicity	No	No
5	Hepatotoxicity	Inactive	Inactive

### 3.2 Extraction and Primary Phytochemical Screening

From the cold maceration we obtained around 4.12% of extractive yield. Primary phytochemical screening confirmed the presence of alkaloids, glycosides, flavonoids, and phenolic compounds in the IQ extract. This extract was stored at 4°C until further use.

### 3.3 Common Targets Between QI and OC

**FIGURE 1:**
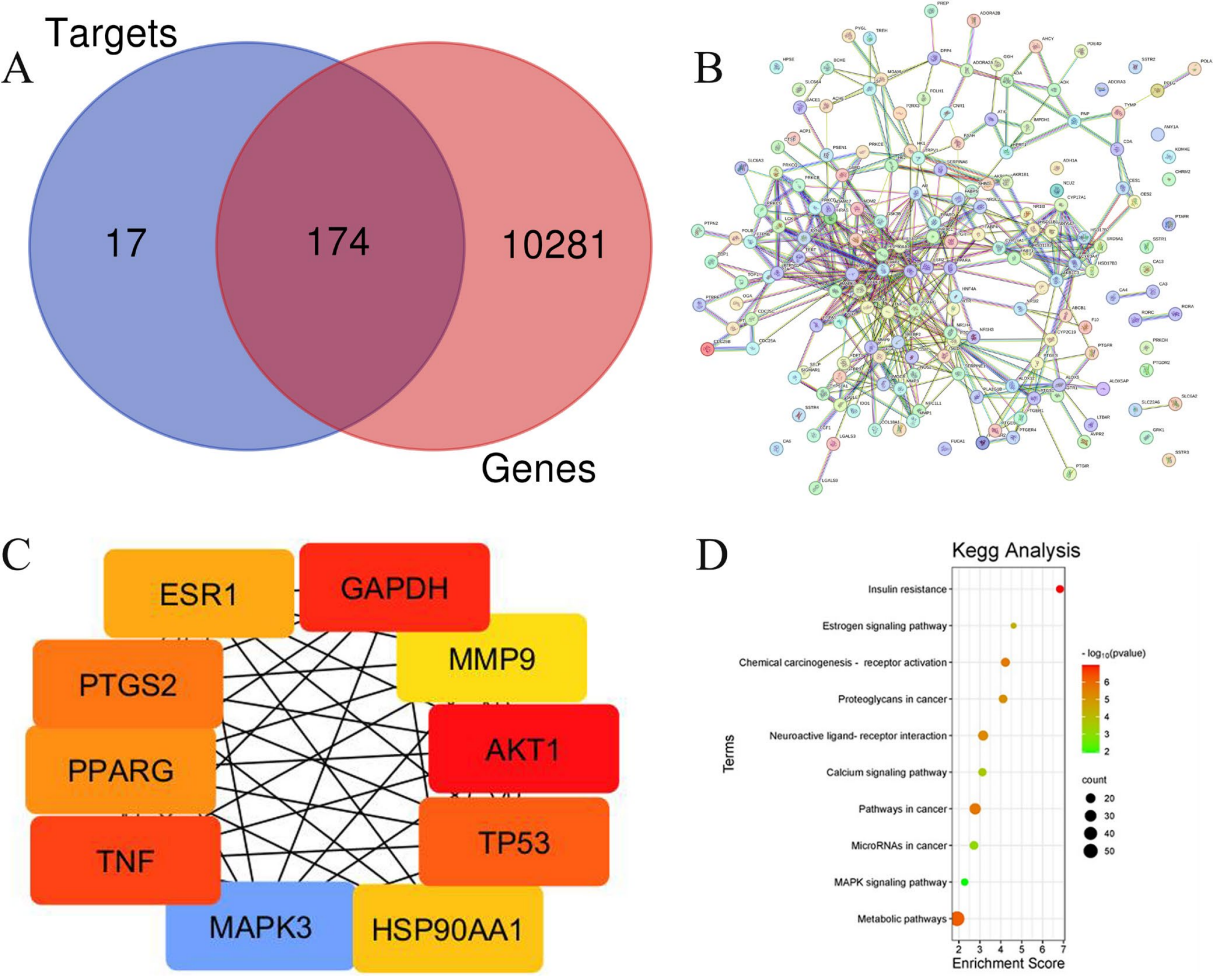
Common gene Venn diagram (A), PPI (B), top 10 gene interactions (C), and the top 10 significant signaling pathways associated with QI and OC related common targets (D). The bubble chart uses color coding to represent −log (*p*‐values), with red indicating the highest statistical significance.

We selected 2 phytoconstituents on the basis of provided inclusion criteria (in method section). A total of 201 targets were found from SwissTargetPrediction and Stitch database. Duplicate targets were removed, and the remaining unique 191 targets were taken for further analysis. Similarly, 10,457 genes related to oral cancer were obtained from GeneCards and DisGeNET. Network pharmacology analysis was conducted to elucidate the potential molecular mechanisms underlying the anticancer activity of *Q. infectoria* against oral cancer. A total of 174 common genes between 10,455 oral cancer‐associated genes and 191 compounds related targets were identified, as illustrated in the Venn diagram (Figure 1A). These 174 intersecting genes were considered potential therapeutic targets through which *Q. infectoria* may exert its pharmacological effects.

### 3.4 Protein–Protein Interaction (PPI)

The string database was used to visualize the common targets associated with QI and OC. The PPI network constructed using these targets (Figure 1B) revealed a complex and highly interconnected topology, indicating strong functional associations among the target proteins. Additionally, the Cytohubba plugin for the Cytoscape program predicted hub genes, and the degree approach was used to select the top 10 genes. Further topological analysis identified key hub genes including GAPDH, AKT1, TP53, TNF, ESR1, PTGS2, PPARG, MAPK3, HSP90AA1, and MMP9 (Figure 1C), which are critically involved in inflammation, cell cycle regulation, apoptosis, and signal transduction—processes known to play significant roles in oral carcinogenesis. Insulin resistance shows the strongest association (−log_10_ (*p* value) > 6), with a high enrichment score, followed by estrogen signaling and chemical carcinogenesis pathways (Figure 1D). These pathways, marked by large red and orange bubbles, reflect key biological processes enriched in the study, emphasizing their relevance to the analyzed genes.

### 3.5 KEGG and GO Enrichment Analysis

KEGG pathway enrichment analysis (Figure 1D) revealed that the intersecting targets were significantly enriched in pathways related to insulin resistance, estrogenic signaling, chemical carcinogenesis‐receptor activation, calcium signaling, and the MAPK pathway, as well as several cancer‐associated and metabolic pathways. Notably, the MAPK signaling pathway and pathways in cancer were among the most enriched, indicating that *Q. infectoria* may modulate multiple signaling cascades involved in tumor progression and survival. Collectively, these findings highlighted the multi‐targeted and pathway‐oriented mechanisms of *Q. infectoria* , supporting its therapeutic potential as a complementary strategy for the treatment of oral cancer.

**FIGURE 2:**
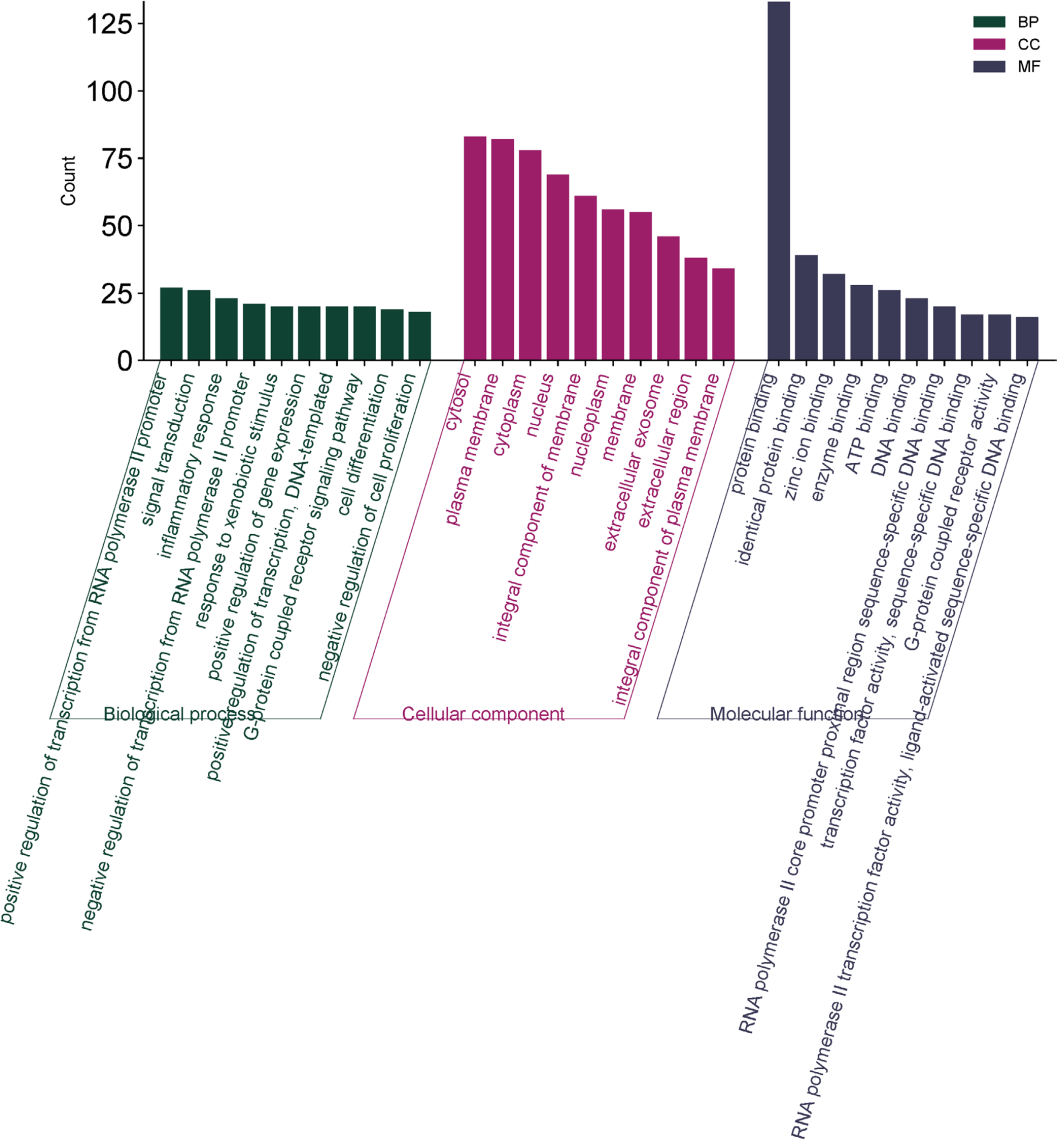
The top 10 significant GO enrichment terms which include biological process, cellular components, and molecular functions associated with QI and OC related common targets.

To further understand the functional roles of the 174 overlapping targets between QI and OC, Gene Ontology (GO) enrichment analysis was performed, categorized into Biological Process (BP), Cellular Component (CC), and Molecular Function (MF) (Figure 2). About 655 GO items were collected, 453 of which were BP, 59 CC, and 143 MF. The top 10 enriched GO terms from each category were identified. Under the BP category (green bars), the most significant terms included positive and negative regulation of transcription from RNA polymerase II promoter, inflammatory response, and signal transduction, indicating that QI targets are heavily involved in the transcriptional regulation and immune response mechanisms associated with oral cancer progression.

In the CC category (Figure 2, purple bars), the targets were predominantly localized to the cytoplasm, nucleus, cytosol, and plasma membrane, with notable enrichment in components such as the integral component of membrane and extracellular region. These findings suggest that the active targets function in diverse cellular compartments and may influence membrane receptor signaling, intracellular signaling cascades, and nuclear transcriptional machinery.

Regarding MF (blue bars), key enriched functions included protein binding, zinc ion binding, enzyme binding, and transcription factor binding, suggesting a wide range of molecular interactions. Particularly, terms such as RNA polymerase II core promoter binding and sequence‐specific DNA binding highlight the importance of transcriptional control in the mechanism of QI against oral cancer. Overall, these GO annotations reinforce the multifaceted molecular mechanisms by which QI may modulate oncogenic pathways and support its potential as a therapeutic agent.

### 3.6 Protein Validation

**FIGURE 3:**
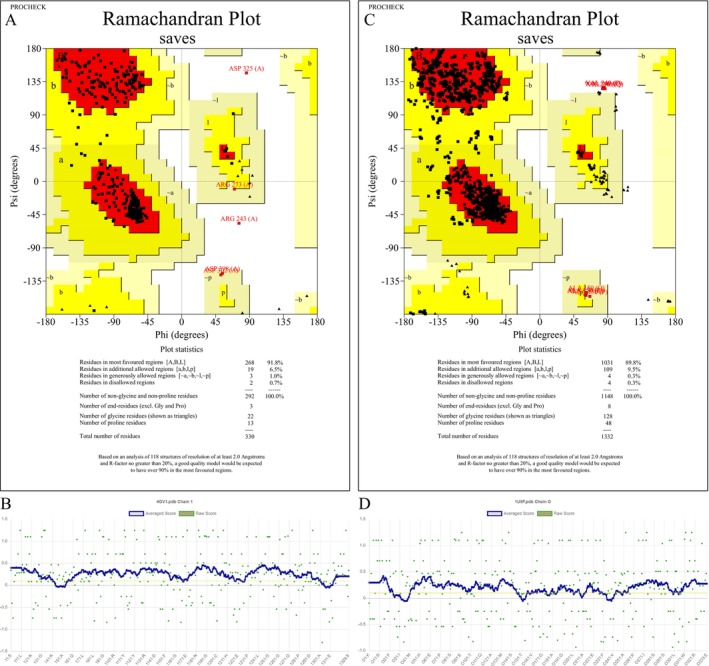
Ramachandran plot of protein 4GV1 (A). The plot displays the distribution of backbone dihedral angles (Φ and Ψ) of the protein residues. Most residues are clustered within the energetically favorable regions (red), indicating a high‐quality model. Verify3D structure of 4GV1. It passed the 3D analysis. 90.27% of the residues have averaged 3D‐1D score ≥ 0.1 (B). Ramachandran plot of protein 1U8F (C). Verify3D structure of 1U8F. It passed the 3D analysis. 82.06% of the residues have averaged 3D‐1D score ≥ 0.1 (D).

The structural validation of the modeled proteins 4GV1 and 1U8F was carried out using Ramachandran plot analysis and Verify3D assessment. The Ramachandran plot for 4GV1 revealed that 91.3% of the residues were located in the most favored regions, 6.1% in additionally allowed regions, 1.0% in generously allowed regions, and only 0.7% in disallowed regions, indicating a high‐quality model with well‐defined backbone conformations. In contrast, the Ramachandran plot for 1U8F showed 90.3% of residues in the most favored regions, 6.2% in additionally allowed regions, 0.3% in generously allowed regions, and 3.1% in disallowed regions (Figure 3). Although both models exhibited acceptable stereochemical quality, 4GV1 demonstrated slightly better backbone geometry with fewer outliers. Furthermore, the Verify3D analysis confirmed that both models passed the 3D‐1D compatibility test. For 4GV1, 90.27% of the residues had an average 3D‐1D score ≥ 0.1, while 1U8F showed 82.06% of residues above this threshold. These results supported the overall structural reliability of both models, with 4GV1 showing superior stereochemical and structural validation scores.

### 3.7 Molecular Docking Analysis

**FIGURE 4:**
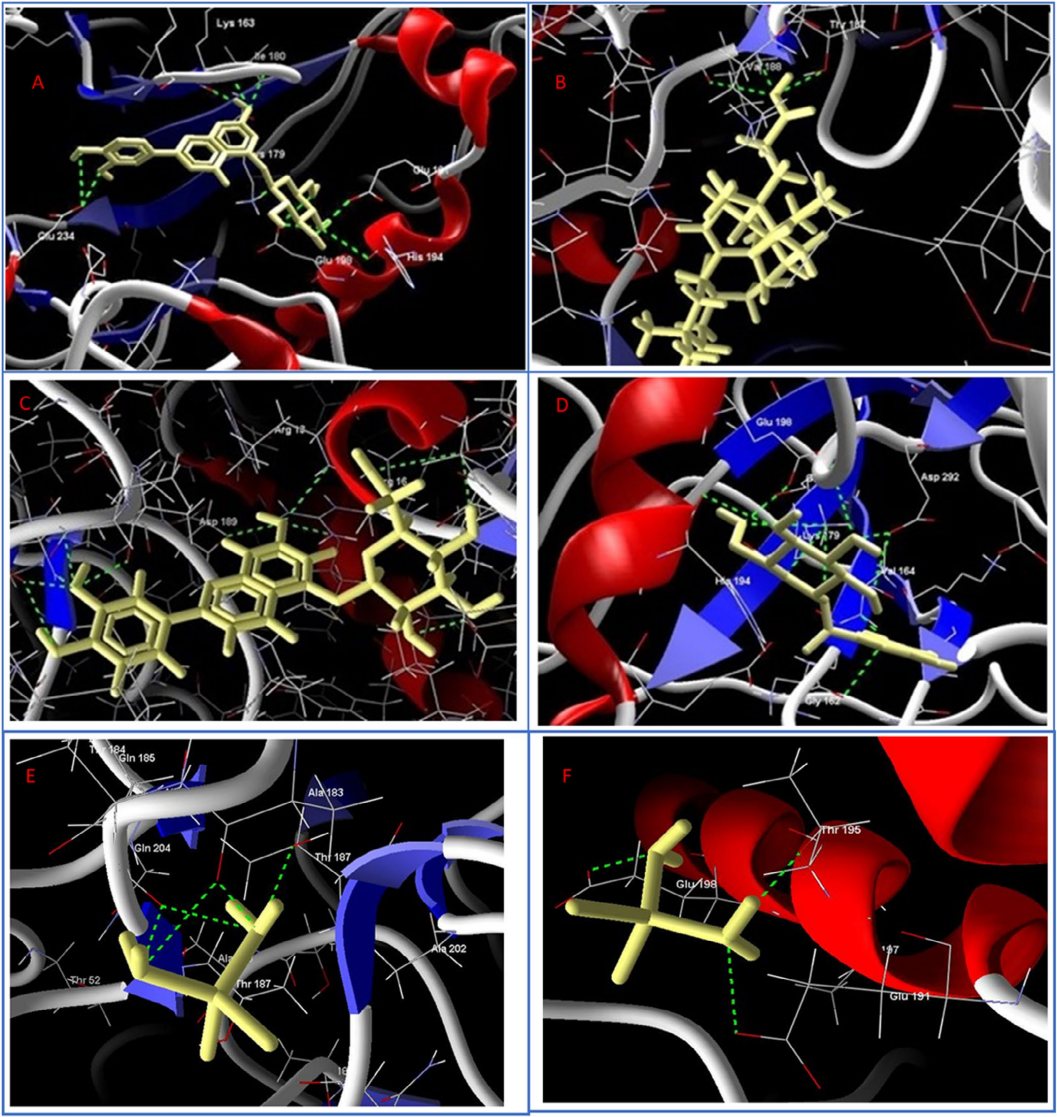
Docking pose of standard compound Galuteolin within the AKT1 binding pocket (4GV1), showing hydrogen bonds with key residues Lys163, Lys179, Glu198, and Glu234, indicating stable interaction through electrostatic and polar contacts (A). Nyctanthic acid docked with GAPDH (1U8F), forming hydrogen bonds primarily with Tyr138 and Arg198, suggesting a stable polar interaction in the active site (B). Standard Galuteolin docked into the active site of GAPDH (1U8F), forming interactions with residues Glu198 and Asp292, stabilizing the ligand in the catalytic groove (C). Nyctanthic acid in complex with AKT1 (4GV1), interacting notably with Arg78, Phe161, Glu198, and Asp189 through a combination of hydrogen bonds and hydrophobic interactions, indicating strong binding stability (D). Molecular docking interaction of Cisplatin against 1U8F (E) and 4GV1 (F).

To gain insights into the binding affinities and molecular interactions of *Quercus infectoria* active constituents against key oral cancer‐associated targets, molecular docking studies were performed using Nyctanthic acid and β‐Glucogallin. The top‐ranked hub genes AKT1 and GAPDH, identified from the network pharmacology analysis, were selected as target proteins. The corresponding crystal structures of AKT1‐related protein (PDB ID: 4GV1) and GAPDH‐related protein (PDB ID: 1U8F) were retrieved from the RCSB Protein Data Bank. The best binding score was selected for analysis. We employed a rigid receptor–flexible ligand approach to simulate binding interactions between selected *Quercus infectoria* phytoconstituents and target proteins. Only ligand‐protein complexes with binding energies lower than −6.0 kcal/mol were considered potentially significant, based on widely accepted thresholds for biologically relevant interactions. The molecular docking analysis revealed that Nyctanthic acid exhibited the most favorable binding affinity toward the AKT1 protein, with a binding affinity of −125.426 kcal/mol. This score was superior to both β‐Glucogallin (−96.7558 kcal/mol) and the reference flavonoid Galuteolin (−123.705 kcal/mol), indicating a stronger and potentially more strong interaction of Nyctanthic acid with the AKT1 binding pocket (Figure 4D, Table 4).

**TABLE 4:** Details of calculated molecular properties of β‐Glucogallin and Nyctanthic acid.

Compounds	AKT1 (PDBID: 4GV1)		GAPDH (PDBID: 1U8F)	
	Binding affinity	Amino acids involved in interaction	Binding affinity	Amino acids involved in interaction
Β‐Glucogallin	−96.7558	Thr160, Phe161	−112.534	Thr187, Ala183, Gln185, Lys186, Val188
Nyctanthic acid	−125.426	Gly162, Val164, Asp292, Gly 94, His194, Glu198, Lys179	−113.841	Val188, Thr187
Galuteolin (reference)	−123.705	Lys179, Glu198, Glu191, His194, Glu234, Lys163, Ile180	−117.778	Gln185, Ala183, Arg13, Ser98, Thr187, Asp35, Arg16, Asp189
Cisplatin (reference)	−103.1824	Glu191, Thr195, Glu198	−102.8101	Ala183, Thr187, Gln204

Against GAPDH (1U8F), Nyctanthic acid also showed a competitive binding affinity (−113.841 kcal/mol) compared to β‐Glucogallin (−112.534) and Galuteolin (−117.778 kcal/mol), highlighting its comparable affinity toward this cancer‐related protein target. The docking interaction of Galuteolin with 4GV1 and 1U8F served as positive controls, validating the reliability of the docking protocol. Galuteolin demonstrated strong interaction with multiple residues in AKT1, including Lys179, Glu198, Glu191, His194, Glu234, Lys163, and Ile180, implying high binding affinity and stability (Figure 4A). In GAPDH, it engaged with Gln185, Ala183, Arg13, Ser98, Thr187, Asp35, Arg16, and Asp189, reflecting widespread contacts likely across different regions of the binding pocket (Figure 4C). Key hydrogen bonding and hydrophobic interactions were observed between Nyctanthic acid and critical residues such as Glu198, Gly162, Asp292, His194, and Lys179 in AKT1, and Tyr 187 and Val188 in GAPDH, contributing to the high binding affinity (Figure 4B). The reference drug cisplatin showed binding affinity of −103.1824 and −102.8101 kcal/mol against AKT1 (4GV1) and GAPDH (1U8F) proteins respectively. Cisplatin interacted with fewer residues—Glu191, Thr195, and Glu198 in AKT1, and Ala183, Thr187, and Gln204 in GAPDH—suggesting a more limited but possibly precise binding mode (Figure 4E,F).

These interactions suggest potential inhibitory effects on AKT1 and GAPDH activity, which are involved in cancer cell survival and metabolic pathways, respectively. The results reinforce Nyctanthic acid as a promising multi‐target lead compound from *Q. infectoria* , with the capability to modulate key oncogenic proteins involved in oral cancer pathogenesis.

The RMSD value from re‐docking analysis was found to be < 2 Å, which confirms that the docking algorithm and scoring functions employed (Binding affinity in Molegro Virtual Docker) can reliably predict ligand binding conformations. Consequently, the docking results for Nyctanthic acid and β‐Glucogallin were interpreted with high confidence regarding their binding orientations and affinities.

### 3.8 MD Simulation

#### 3.8.1 Protein‐Ligand Stability

**FIGURE 5:**
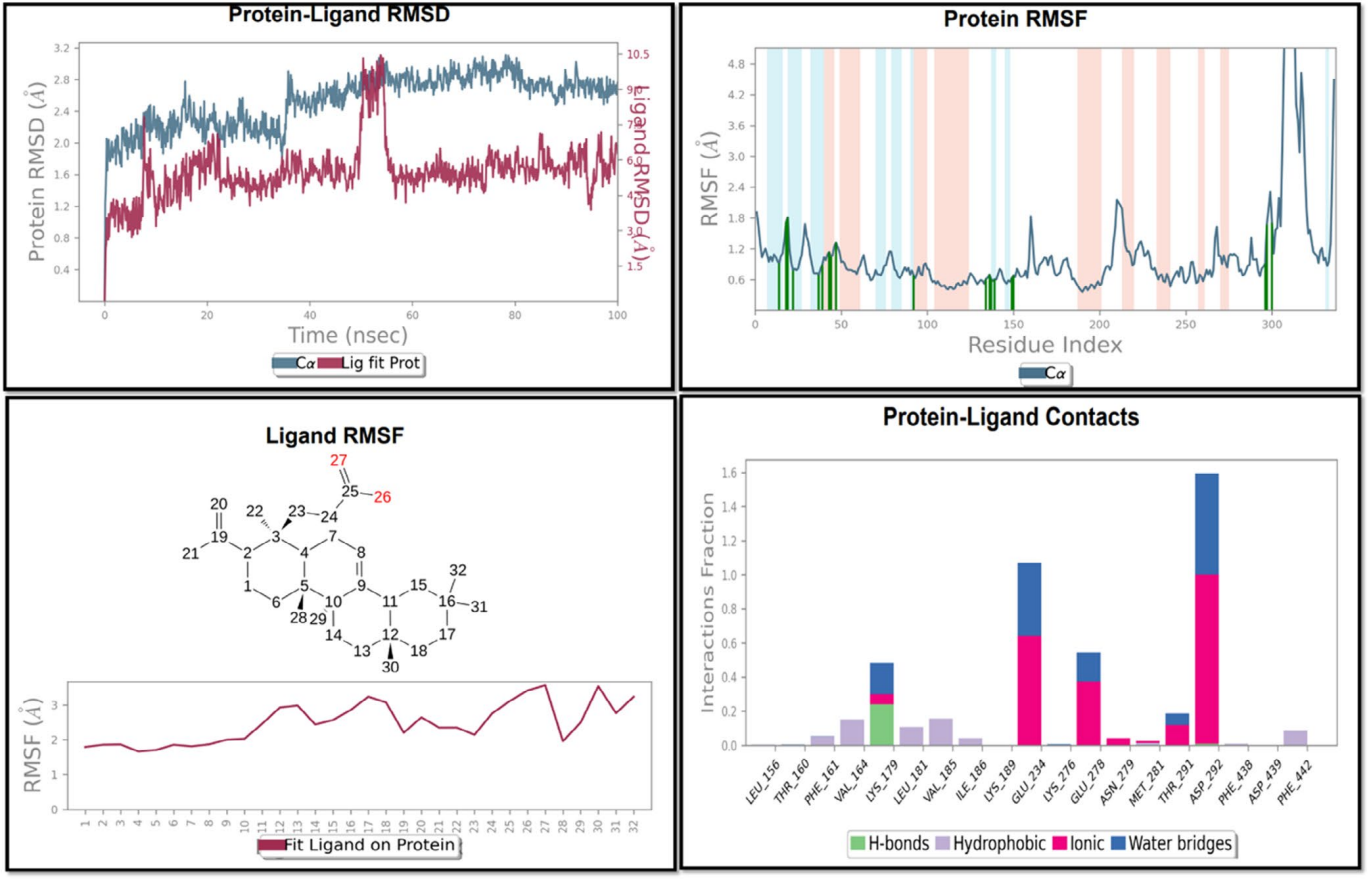
MD simulation analysis of protein–ligand complex. The RMSD plots show structural stability of protein and ligand. The protein RMSF highlights residue flexibility. The ligand RMSF is mapped on its structure. The key protein–ligand interactions show strong binding via hydrogen bonds, hydrophobic, ionic, and water bridge interactions.

To validate the stability and interaction dynamics of the docked Nyctanthic acid–AKT1 complex, a 100 ns MD simulation was carried out. The RMSD plot (Figure 5) revealed that the Cα backbone of the AKT1 protein maintained moderate stability throughout the trajectory, fluctuating within the range of 1.8–2.7 Å, indicating a well‐equilibrated protein conformation. Meanwhile, the ligand RMSD remained below 4.1 Å, suggesting that Nyctanthic acid remained moderately bound within the binding pocket with some displacement, confirming a decent interaction over time. This binding behavior is biologically significant, as it reflects the ability of Nyctanthic acid to remain anchored at the active site—an essential trait for sustained inhibition of AKT1 signaling in cancer cells.

The RMSF analysis of the AKT1 residues (Figure 5) showed minor fluctuations (< 2.0 Å) for the majority of residues, with notable peaks observed in the loop regions and terminal ends, particularly near residues around the 50–80 and 300–320 indices. These fluctuations are typical in flexible loop regions and did not affect the overall structural integrity of the protein. The highlighted interaction regions further confirmed the ligand's influence on localized residue motion. The ligand RMSF plot (Figure 5) provided insight into the flexibility of Nyctanthic acid atoms during the simulation. Most of the atoms displayed low RMSF values (1.05 Å), with slight flexibility observed at atoms 26, 27, and 30, possibly due to their peripheral positioning and limited engagement in stable interactions. This supports the conclusion that the core of Nyctanthic acid remained rigid, contributing to its firm binding conformation.

#### 3.8.2 Protein‐Ligand Interaction Histogram

The protein–ligand interaction profile (Figure 5) highlighted frequent and stable contacts between Nyctanthic acid and key AKT1 residues, including Val164, Lys179, Leu181, Lys189, Lys276, and Asp292. Notably, among the residues analyzed, Asp292 stands out prominently with the highest overall interaction fraction (> 1.5), predominantly through ionic interactions and water bridges, indicating a strong and stable electrostatic interaction with Nyctanthic acid. Additional stabilizing roles are evident for GLU234, GLU278, and THR291, all of which display significant ionic or hydrogen bonding contributions. These multiple, concurrent noncovalent interactions contribute to the biological activity of Nyctanthic acid by promoting strong and sustained inhibition of AKT1—a kinase heavily implicated in oral cancer pathogenesis via the PI3K‐Akt pathway.

#### 3.8.3 Compactness and Surface Analysis

**FIGURE 6:**
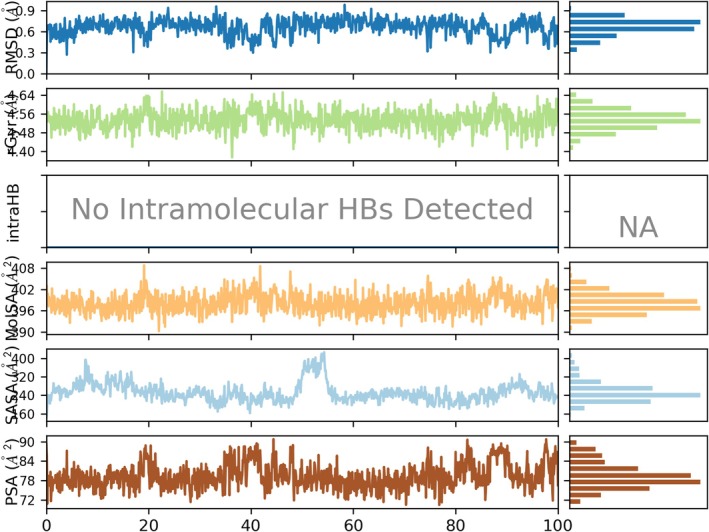
Plots of ligand properties (RMSD, rGyr, intraHB, MolSA, SASA, PSA). It illustrates various ligand properties over the 100 ns molecular dynamics (MD) simulation, providing a comprehensive understanding of the ligand's conformational stability and dynamic behavior within the binding pocket.

The ligand properties analysis of Nyctanthic acid during its 100 ns molecular dynamics simulation with AKT1 (PDB ID: 4GV1) reveals stable and consistent behavior, affirming its potential as a bioactive candidate. The RMSD of the ligand remained in the range of approximately 0.6–1.0 Å, indicating that Nyctanthic acid maintained a stable conformation and orientation within the active site of AKT1 throughout the simulation (Figure 6). This low RMSD variability is a strong indication of the ligand's ability to retain its binding pose without exhibiting significant fluctuations or detachment from the protein.

The radius of gyration (rGyr), which reflects the compactness of the ligand structure, remains relatively stable throughout the simulation, fluctuating narrowly around 4.60 Å. This consistent rGyr value indicates that the ligand maintains a tightly folded conformation and does not undergo significant structural expansion or collapse. Interestingly, no intra‐molecular hydrogen bonds (IntraHB) were detected during the entire simulation period, suggesting that the ligand's structural integrity is not dependent on internal hydrogen bonding. Instead, its stability is likely reinforced through persistent interactions with the surrounding protein residues and solvent environment. The molecular surface area (MolSA) displays only minor fluctuations in the range of 396–408 Å^2^, supporting the notion of a structurally stable ligand with minimal conformational drift.

Furthermore, the solvent‐accessible surface area (SASA) remains generally stable within the range of 340–400 Å^2^, with a noticeable but temporary increase peaking around 420 Å^2^ at approximately the midpoint of the simulation (50 ns). This transient rise may reflect a short‐lived outward shift or reorientation of the ligand, which quickly stabilizes back to its prior state—highlighting the dynamic yet resilient nature of the ligand‐protein complex. The polar surface area (PSA), an indicator of polar functional group exposure to the solvent, also remains within a moderate range of 72–96 Å^2^, indicating that polar interactions are maintained consistently throughout the trajectory. Taken together, these parameters demonstrate that the ligand preserves its structural compactness and surface characteristics, despite minor dynamic fluctuations, and remains conformationally stable in the active site. This stability reinforces the ligand's potential as a promising candidate for therapeutic development.

Further, the molecular surface area (MolSA) remained relatively constant (398.4 Å^2^), indicating steady exposure to the protein's binding environment (Figure 6, Table 5). The solvent‐accessible surface area (SASA) showed a minor and transient increase between 60 and 70 ns, which could suggest a brief conformational rearrangement. However, the ligand quickly returned to a stable SASA range (380–400 Å^2^), confirming that this fluctuation did not compromise its overall stability. Similarly, the polar surface area (PSA) showed moderate variation (72–96 Å^2^), which reflects the ligand's balanced polarity—favorable for sustaining interactions with the polar residues in AKT1's binding site.

**TABLE 5:** Summary of molecular dynamics parameters (mean ± SD) for the protein–ligand complex over 100 ns simulation.

Property	Min	Max	Average ± SD
Protein RMSD (Å)	1.8	2.7	2.3 ± 0.18
Ligand RMSD (Å)	3.0	9.7	4.1 ± 0.10
Ligand RMSF (Å)	0.4	1.7	1.05 ± 0.25
Rg (Å)	4.45	4.68	4.52 ± 0.05
MolSA (Å^2^)	390	408	398.4 ± 4.2
SASA (Å^2^)	240	400	260.1 ± 10.3
PSA (Å^2^)	72	96	82.7 ± 5.1

The secondary structure analysis of the target protein, as illustrated in Figure S1, reveals important insights into its structural stability during the 100 ns molecular dynamics simulation. The protein exhibits a well‐organized secondary structure, with 26.52% α‐helices and 14.39% β‐strands, contributing to a total secondary structure content of 40.91%. These structured regions are distributed along the protein sequence and remain stable throughout the simulation, indicating that the protein maintains its native‐like fold under the simulated conditions. The presence of well‐defined helices and strands suggests a rigid core that supports structural integrity, particularly around the ligand‐binding region. In contrast, the unstructured regions, likely comprising loops and coils, may offer the necessary flexibility for ligand recognition and binding. Overall, the consistent distribution of secondary structure elements supports the structural robustness of the protein and reinforces the validity of the protein‐ligand interaction analysis carried out in this study.

Figure S2 presents the secondary structural residue profile of the target protein over the 100 ns molecular dynamics simulation, providing a time‐resolved view of the stability and transitions of secondary structural elements (SSEs). The top panel of the figure shows the overall percentage of residues adopting secondary structure elements—such as α‐helices and β‐strands—throughout the simulation. It remains consistently around 40%–45%, indicating that the protein maintains a stable secondary structure under simulation conditions. The bottom panel maps the secondary structure assignment of each residue across time, with α‐helices represented in blue and β‐strands in red, while unstructured regions such as loops or coils appear white. The dense and uninterrupted bands of red and blue across specific residue indices suggest the persistence of structured regions, particularly in the core of the protein. These stable regions likely play critical roles in maintaining the protein's overall fold and supporting ligand binding. The presence of consistent SSEs throughout the simulation trajectory reinforces the structural robustness of the protein and validates its suitability for dynamic interaction studies with the ligand.

The schematic highlights a water‐mediated hydrogen bond between the ligand and the negatively charged residue Asp292, which is part of chain A of the protein (Supporting Information, Figure S3). This interaction is observed in approximately 30% of the total simulation frames, meaning that in 30 out of every 100 frames sampled during the trajectory, a consistent contact is maintained between the ligand and Asp292 via a bridging water molecule. The percentage was calculated based on the number of frames in which the donor‐acceptor distance and angle criteria for a hydrogen bond were met, as determined by the protein‐ligand contact analysis module in the simulation software. The persistence of this interaction—especially in the absence of multiple competing contacts—suggests that Asp292 plays a crucial role in stabilizing the ligand within the binding pocket. Although transient, such interactions maintained over a significant portion of the simulation time are considered prominent due to their potential contribution to the ligand's binding affinity and biological activity. Therefore, the interaction with Asp292 is not only recurrent but may also be functionally relevant to the protein‐ligand binding mechanism.

### 3.9 DFT Analysis

Structure optimization of molecular systems can be effectively performed by DMol3, a quickest method for molecular DFT computations using delocalized internal coordinates. Numerous energies have been estimated using the technique, including dipole magnitude, HOMO and LUMO energies, total energy, and binding energy. By default, 0.03 is the electron density isovalue. There are two phases to molecular orbitals: the negative phase, which is red, and the positive phase, which is blue and has an isovalue of 0.01.

**FIGURE 7:**
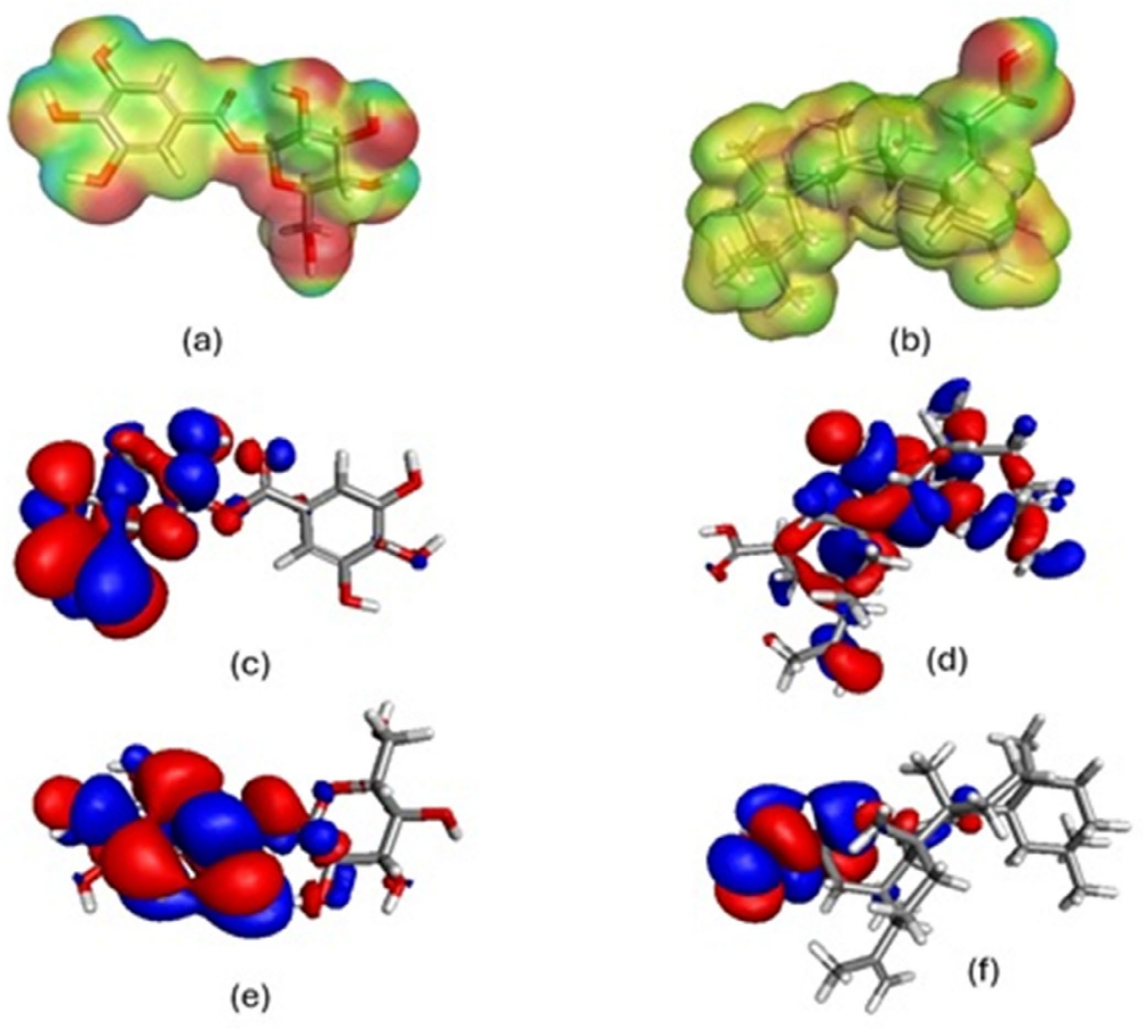
(a) Surface plots showing the electrostatic potential mapped on the isosurface of the electron density of β‐Glucogallin (b) Surface plots showing the electrostatic potential mapped on the isosurface of the electron density of Nyctanthic acid (c) Visualization of the HOMO molecular orbitals of β‐glucogallin (d) Visualization of the HOMO molecular orbitals of Nyctanthic acid (e) Visualization of the LUMO molecular orbitals of β‐Glucogallin (f) Visualization of the LUMO molecular orbitals of Nyctanthic acid.

In Figure 7, a comprehensive pictorial representation is provided. Figures 7a and 7b the electrostatic potential on the isosurface of the electron density of β‐Glucogallin and Nyctanthic acid, respectively. The HOMO molecular orbitals of Nyctanthic acid are visualized in Figure 7d, whereas those of β‐Glucogallin are displayed in Figure 7c. Figure 7e depicts the β‐Glucogallin LUMO molecular orbitals, while Figure 7f shows the Nyctanthic acid LUMO orbitals. The total energy of β‐Glucogallin is −1247.35, while that of Nyctanthic acid is −1310.55. β‐Glucogallin and Nyctanthic acid have binding energies of −14.7953 and −7.5022, respectively (Table 6). The HOMO and LUMO energies for β‐Glucogallin were determined to be −0.209401 and −0.100057, respectively, while those for Nyctanthic acid were determined to be −0.187304 and −0.0397976. Nyctanthic acid and β‐Glucogallin were found to have dipole magnitudes of 0.571208 and 2.03194.

**TABLE 6:** DFT results of β‐Glucogallin and Nyctanthic acid.

SN	Compound	Total_energy_DMol3	Binding_energy_DMol3	HOMO_energy_DMol3	LUMO_Energy_DMol3	Dipole_Mag_DMol3
1	β‐Glucogallin	−1247.35	−7.5022	−0.209401	−0.100057	2.03194
2	Nyctanthic acid	−1310.55	−14.7953	−0.187304	−0.0397976	0.571208

### 3.10 In Vitro Validation of Cytotoxicity Analysis

The cytotoxic effect of QI extract and the reference drug Cisplatin was evaluated on KB oral cancer cells using the MTT assay. Both agents induced a dose‐dependent reduction in cell viability over a concentration range of 31.25–500 μg/mL. Untreated control cells showed normal proliferation, confirming assay validity. QI extract exhibited an IC_50_ value of 224.41 ± 2.01 μg/mL, indicating substantial cytotoxic activity, though slightly lower than that of Cisplatin, which had an IC_50_ of 213.77 ± 1.98 μg/mL (Table 7).

**TABLE 7:** Cell viability andIC_50_ values of QI extract and standard oral cancer cell lines (KB mouth cell line).

Conc. (μg/mL)	% Cell viability		*p*‐value	Significance
	QI extract (SD)	Standard drug cisplatin (SD)		
31.25	80.13 ± 1.03	82.42 ± 1.38	0.062	NS
62.5	79.08 ± 2.07	81.89 ± 1.29	0.051	NS
125	77.29 ± 1.54	80.47 ± 0.85	0.018	*
250	78.11 ± 1.93	81.02 ± 2.12	0.038	*
500	75.12 ± 2.11	79.77 ± 1.20	0.022	*
IC_50_ (μg/mL)	224.41 ± 2.01	213.77 ± 1.98	0.005	**

Abbreviation: NS = not significant.

* *p* < 0.05.

** *p* < 0.01.

**FIGURE 8:**
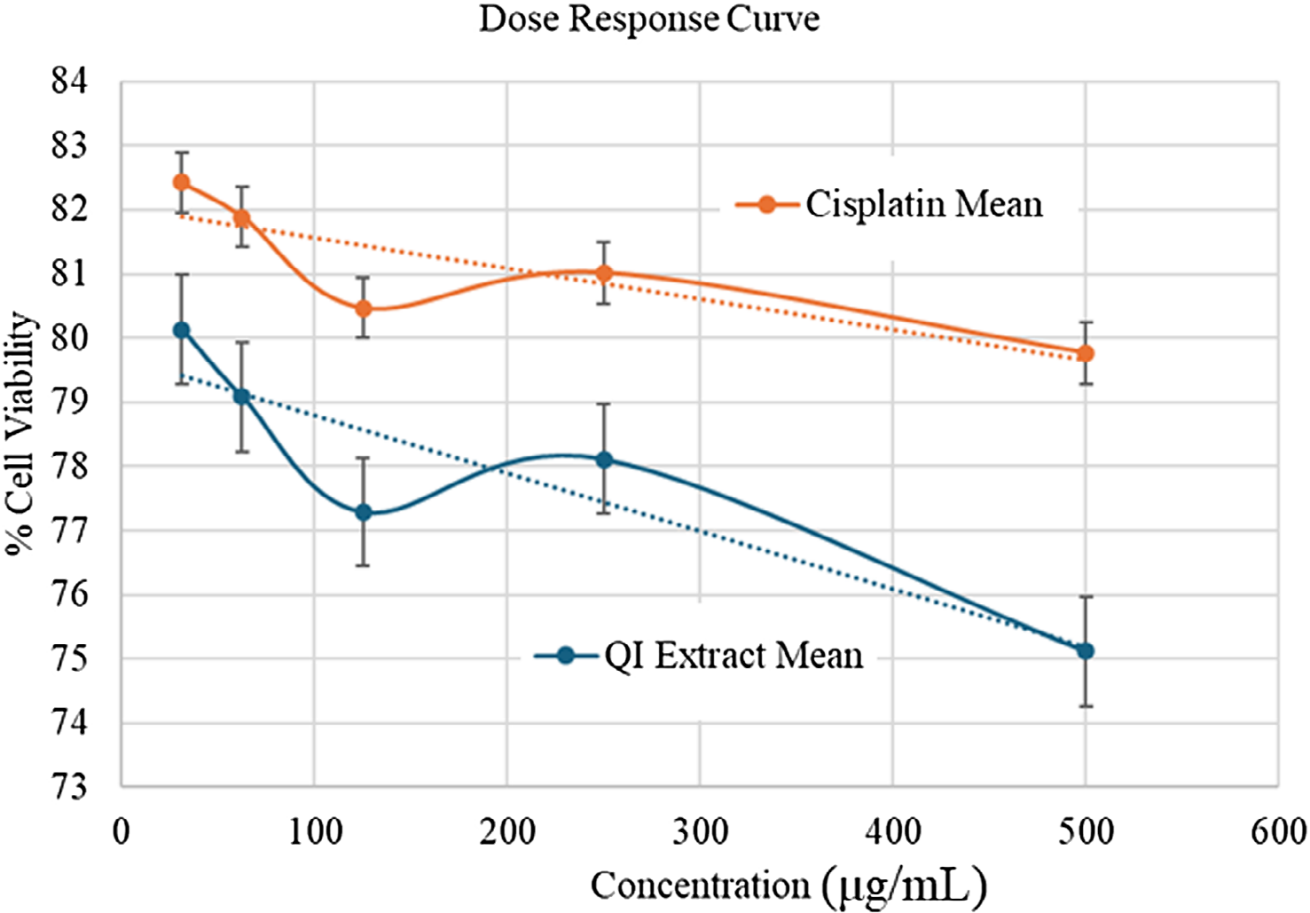
Dose–response curve and error bar of extract and reference Cisplatin.

Statistical analysis by unpaired t‐test revealed that at lower concentrations (31.25 and 62.5 μg/mL), there was no significant difference between QI extract and cisplatin in terms of cell viability (*p* > 0.05). However, at concentrations 125 μg/mL and above, a statistically significant difference (*p* < 0.05) was observed, suggesting enhanced cytotoxic efficacy of the standard drug compared to the extract. A highly significant difference (*p* < 0.01) was noted between the IC_50_ values of the two groups. Additionally, the dose–response curve demonstrated a concentration‐dependent decrease in cell viability for both the QI extract and Cisplatin in KB oral cancer cells (Figure 8). QI extract exhibited a steeper decline in viability compared to Cisplatin, indicating higher cytotoxicity at equivalent doses.

## 4 Discussion

This study integrates a comprehensive multi‐layered approach to validate the anticancer potential of *Q. infectoria* against oral cancer. Network pharmacology laid the foundation by identifying key targets and pathways involved in cancer progression, highlighting the polypharmacological nature of QI phytoconstituents. The enrichment of genes involved in apoptosis and signaling pathways aligns with the phytomedicinal role of QI in traditional anti‐inflammatory and anti‐cancer remedies. Clinical studies have shown that AKT1 overexpression correlates with poor prognosis and resistance to conventional treatments such as cisplatin and radiotherapy. Similarly, GAPDH, beyond its role in glycolysis, is overexpressed in many cancers including OC, where it contributes to the Warburg effect, supports rapid energy production, and modulates apoptosis and gene transcription. AKT inhibitors like Ipatasertib show efficacy but are limited by toxicity and resistance. Furthermore, most current therapies do not simultaneously address metabolic reprogramming—A gap that GAPDH inhibition can fill. By targeting both AKT1 and GAPDH, Nyctanthic acid may offer a more comprehensive therapeutic approach, potentially improving outcomes in oral cancer where resistance and recurrence remain major clinical challenges. These findings support further preclinical evaluation of Nyctanthic acid as a targeted, multi‐pathway oral cancer therapeutic.

### 4.1 Mechanistic Insights

Network pharmacology analysis identified AKT1 (serine/threonine protein kinase 1) and GAPDH as key hub genes involved in OC, with AKT1 playing a crucial role in the PI3K/Akt signaling pathway, which is often dysregulated in cancers. AKT1 plays a pivotal role in the PI3K/AKT/mTOR signaling pathway, which regulates crucial cellular processes including cell survival, proliferation, angiogenesis, and metabolism (Glaviano et al. 2023). Additionally, it promotes cell survival by inhibiting apoptosis, supports uncontrolled proliferation through mTOR activation, enhances angiogenesis via vascular endothelial growth factor (VEGF) upregulation, and facilitates metastasis by regulating matrix metalloproteinases and epithelial–mesenchymal transition (Versari et al. 2025). Hyperactivation of AKT1 is commonly observed in various malignancies, including OC, and contributes to tumor cell resistance to apoptosis and enhanced invasiveness. Targeting AKT1 has been proposed as a promising strategy for reversing chemoresistance and suppressing tumor growth in OC (Rajendran et al. 2024). GAPDH, traditionally known for its role in glycolysis, has been recognized as a multifunctional protein in cancer, involved in transcriptional regulation, apoptosis, and cellular redox balance. Its overexpression has been reported in several cancers, including OC, where it enhances tumor cell metabolism and supports oncogenic transformation. Inhibiting GAPDH can disrupt cancer metabolism and trigger apoptotic pathways (Butera et al. 2019; Liberti et al. 2017). Furthermore, KEGG and GO enrichment analysis highlighted crucial pathways and the involvement of common QI–OC targets. In a study, in vitro assay showed that QI gall extract can suppress the growth of A549, BGC823, and KYSE‐30 cells by inducing apoptosis, and such an effect may be mediated through the mitochondria‐dependent pathway (Tofigh et al. 2024).

Molecular docking studies revealed that Nyctanthic acid exhibited a higher binding affinity to AKT1 (Binding affinity: −125.426) compared to β‐Glucogallin (−96.7558) and the reference compound Galuteolin (−123.705). To rationalize these findings, it is important to compare them with known AKT1 inhibitors. For instance, MK‐2206, a well‐characterized allosteric AKT1 inhibitor currently in clinical trials, has demonstrated binding energy around −8.83 kcal/mol (dock score = −26.55) in a molecular docking study. They used Dock v.6.5 (University of California, San Francisco) software for docking simulation (Rehan et al. 2014). The natural compound dieckol has shown binding energies of −10.8 kcal/mol with AKT1 and −11.4 kcal/mol with AKT2. They used PyRx v0.8 (AutoDock Vina) to calculate binding energies (Natarajan et al. 2022). Similarly, andropanoside and neoandrographolide, two natural compounds, showed strong anticancer potential by targeting AKT1. Using Discovery Studio 4.5, both demonstrated high binding affinities (−58.78 and −56.48 kcal/mol) and formed 12 and 10 hydrogen bonds with AKT1, respectively. The in vitro assays showed effective inhibition of MG63 cell viability and AKT1 expression at ~10 μmol/L (Zhong et al. 2022). These values are less negative than the binding affinity observed for Nyctanthic acid in our study, suggesting that Nyctanthic acid may have a stronger binding affinity toward AKT1 compared to these known inhibitors. While Nyctanthic acid showed favorable docking scores, direct experimental or kinetic comparisons are necessary before drawing conclusive comparisons with established inhibitors.

Shile Huang listed the medicinal plant and their natural products responsible for the inhibition of the PI3K/Akt/mTOR signaling pathway in an editorial (Huang 2013). The phytochemical study on *Quercus infectoria* identified several gallotannins, including 6‐O‐digalloyl‐1,2,3,4‐tetra‐O‐galloyl‐β‐D‐glucose, with a strong binding affinity against oral cancer targets such as MMP‐2, NF‐κB p65, and RhoA, showing binding energies up to −10.6 kcal/mol (AutoDock v4.2.6). Furthermore, while *Q. infectoria* tannins rely heavily on multiple hydrogen bonding interactions (up to 8 H‐bonds) and hydrophobic contacts (Ahmad et al. 2024), Nyctanthic acid also forms several stable H‐bonds with crucial residues (e.g., His194, Glu198, Lys179 in AKT1 and Thr187, Val188 in GAPDH). These interactions are consistent with those observed in galuteolin and cisplatin, but Nyctanthic acid's superior binding score underscores its enhanced fit and potential inhibitory effect. While the *Q. infectoria* compounds demonstrate promising interaction profiles against specific cancer‐related signaling proteins, the binding affinities of Nyctanthic acid against AKT1 and GAPDH reflect even more favorable energetics in our selected targets.

**FIGURE 9:**
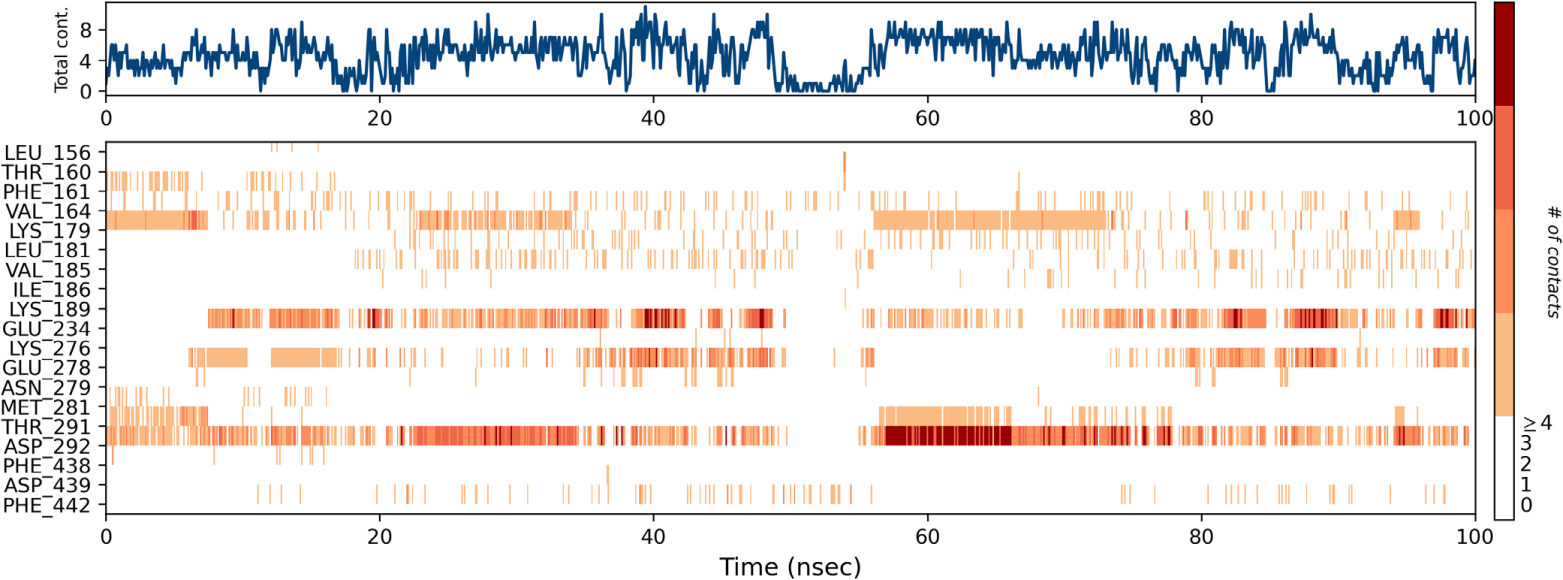
Timeline representation of protein‐ligand contact, it can be deduced that during the course of the simulation run of 100 ns, a number of interactions of amino acids are seen to be formed and broken. Notably, Asp292 consistently maintains a stable interaction across the entire trajectory, indicating its prominent role in ligand binding. This observation is further supported by the protein‐ligand contact plot.

The binding site of AKT1, comprising residues such as Gly157, Lys158, Gly159, Gly162, Lys163, Val164, Ala177, Lys179, Leu181, Met227, Glu228, Ala230, Glu234, Glu278, Met 292, and Phe438, shows varied interaction profiles (Figure S4, Supporting Information). Among the 19 residues analyzed, Asp292 emerges as the most consistently interacting residue across the entire trajectory, maintaining a high contact density, which underscores its role in stabilizing the ligand within the binding pocket. This observation is critical, as Asp292 is also part of the known active site of AKT1 (PDB ID: 4GV1). While residues like Glu234, Lys179, and Glu278 showed moderate and intermittent contact formation, other active‐site residues (Met 227, Ala 230) exhibited negligible contacts, suggesting peripheral involvement or transient binding contributions (Figure 9). The presence of multiple water bridges, especially in Asp292 and Thr291, further indicates solvent‐mediated binding mechanisms, which are common in polar binding pockets.

Furthermore, AKT1 has a critical role in the PI3K‐Akt signaling pathway, which suggests that these results support Nyctanthic acid as a biologically relevant candidate for further development. The docking data, therefore, reinforce the therapeutic potential of Nyctanthic acid in targeting key oncogenic pathways involved in oral cancer progression. MD simulations further confirmed the stability of the Nyctanthic acid–AKT1 complex, suggesting a strong and stable interaction that could inhibit AKT1 activity. The decent RMSD, favorable RMSF, and stable hydrogen bonding profiles of the Nyctanthic acid–AKT1 complex indicated minimal conformational perturbation, reinforcing its suitability as a lead compound. Ligand property metrics such as PSA and MolSA confirmed sustained solvent exposure and conformational compactness, both favorable for bioactivity and cellular absorption.

The integration of network pharmacology with docking, MD simulations, and Density DFT results provides a comprehensive validation of anticancer mechanisms. Network pharmacology identified key targets and oral cancer‐related pathways, which were confirmed by docking studies showing strong binding affinities to these targets. MD simulations further supported the stability of these interactions over time. Additionally, DFT calculations explained the electronic properties of the compounds, correlating their structure with binding affinity to specific targets, such as kinases in the PI3K/Akt pathway. This integrated approach enhanced the understanding of the compounds' mechanisms of action, optimizing their potential as anticancer agents.

### 4.2 Cytotoxic Activity, Quantum Chemical Validation, and Pharmacokinetic Properties

The cytotoxic efficacy observed in the MTT assay corroborated the in silico predictions. Although the QI extract showed a slightly higher IC_50_ than Cisplatin, its dose‐dependent activity and plant‐based origin highlight it as a safer and promising alternative with potential for optimization and reduced side effects. Previous research has demonstrated the cytotoxic effects of QI extracts on various cancer cell lines (Afriza et al. 2024). For instance, an ethyl acetate extract of QI exhibited potent cytotoxic activity against HeLa cervical cancer cells, with an IC_50_ value of 6.33 ± 0.33 μg/mL, and showed cytoselectivity by sparing normal fibroblast (L929) cells (Wan Yusof and Abdullah 2020). Another study reported that QI galls water extract induced apoptosis and autophagic cell death in colorectal cancer cells through the accumulation of intracellular reactive oxygen species (ROS) and modulation of the AKT/mTOR signaling pathway (Kheirandish et al. 2016; Oliveira et al. 2023). QI gall crude extract also showed inhibitory activity against human GBM cancer cells (IC50 = 17.0 μg/mL) (Kamarudin et al. 2021). The anticancer property is related to gallotannin phytoconstituents present in the QI extract (Kamarudin et al. 2021). It is evident that various plant extracts and plant‐based formulations showed good property against oral cancer (Prakash et al. 2021).

In the context of oral cancer, our study is among the first to investigate the effects of QI extract and its constituents on oral cell lines. The MTT assay revealed that the QI extract exhibited dose‐dependent cytotoxicity against KB mouth cancer cells, with an IC_50_ value of 224.41 ± 2.01 μg/mL. Although this IC_50_ value is higher compared to those reported in previous studies on other cancer types, it is important to consider the differences in cancer cell lines and experimental conditions. Notably, the QI extract demonstrated a comparable cytotoxic effect to the standard chemotherapeutic agent cisplatin (IC_50_ = 213.77 ± 1.98 μg/mL), suggesting its potential as an alternative or adjunctive therapy for OC. From a biological efficacy perspective, boiled water extracts of QI demonstrated cytotoxicity exceeding 90% across multiple cancer cell lines (lung, breast, prostate), with IC_50_ values as low as 7.2 μg/mL and a selectivity index (SI) > 4.71 (Jaber 2024). Similarly, the ethyl acetate extract of QI showed a potent IC_50_ of 6.33 ± 0.33 μg/mL against HeLa cells and selectively spared normal fibroblast cells, suggesting favorable therapeutic selectivity (Wan Yusof and Abdullah 2020). These results are comparable to the IC_50_ of 76.82 μg/mL observed for QI extracts in another study, indicating consistency across experimental platforms. The same study reported that the QI extract caused cell cycle arrest at sub‐G0/G1 and increased early apoptosis (Ahmad et al. 2024). Another study demonstrated broader cytotoxicity of QI gall extract against A549, BGC823, and KYSE‐30 cell lines (IC_50_ ~440–465 mg/mL), along with increased apoptosis and altered expression of CCND1, TP53, BCL2, and BAX genes (Tofigh et al. 2024). Several natural compounds have been previously investigated for their therapeutic potential against oral cancer, particularly phytochemicals such as curcumin, resveratrol, quercetin, and epigallocatechin gallate (EGCG). These compounds exert anticancer effects through various mechanisms including apoptosis induction, inhibition of cell proliferation, and suppression of angiogenesis and metastasis. Curcumin, for instance, has been shown to inhibit proliferation and induce apoptosis in oral squamous cell carcinoma (OSCC) via EGFR pathway suppression and modulation of NF‐κB, AP‐1, and other signaling cascades (Almalki et al. 2023; Nocito et al. 2021). Similarly, resveratrol has demonstrated antiproliferative, antimetastatic, and proapoptotic effects in OSCC cells, including inhibition of adhesion, migration, invasion, and targeting mechanisms linked to ferroptosis and chemoresistance. Its clinical potential likewise remains bounded by low oral bioavailability, leading to strategies such as buccal liposomal formulations to enhance local delivery (Angellotti et al. 2023). In this context, Nyctanthic acid stands out in our study for exhibiting a higher binding affinity with AKT1 (−125.426 kcal/mol) than many other plant‐derived compounds coupled with favorable predicted ADMET and MD stability profiles.

The DFT analysis offered quantum chemical validation, with favorable HOMO–LUMO gaps and energy profiles supporting molecular stability and reactivity (Unsal, Oner, et al. 2025). The binding energies and dipole moments further differentiated the bioactive behavior of Nyctanthic acid and β‐Glucogallin, with the former exhibiting lower dipole and HOMO energy, implying effective electron transfer and favorable biological interactions. The integration of experimental and computational strategies strongly supports the therapeutic potential of QI against oral cancer. Nyctanthic acid, in particular, emerges as a promising lead compound warranting further preclinical and clinical validation.

From the computational study, we have predicted both Nyctanthic acid and β‐Glucogallin compounds as nontoxic. It is critical to address the toxicity and safety profiles of these compounds. Despite their natural origin, phytochemicals are not inherently free from adverse effects. Currently, there is limited literature on the cytotoxicity of Nyctanthic acid and β‐Glucogallin in normal (non‐cancerous) human cell lines, which is a significant gap in validating their therapeutic viability. As toxicity is a central concern in drug discovery and development, future in vitro and in vivo studies should rigorously assess the dose‐dependent cytotoxicity, off‐target effects, and therapeutic index of these compounds using standard toxicity assays (e.g., MTT, LDH release, ROS generation) in normal epithelial and fibroblast cell lines. This will ensure that the anticancer effects observed are not accompanied by undesirable harm to healthy tissues, thereby enhancing the translational relevance of our findings.

Nyctanthic Acid exhibited high oral bioavailability and strong drug‐likeness, indicating good absorption potential. However, its high lipophilicity (Log *p* = 8.43) suggests poor aqueous solubility, which may limit its gastrointestinal absorption and lead to non‐specific tissue accumulation. Although it is not a substrate for P‐glycoprotein and does not inhibit major CYP enzymes (CYP1A2, CYP2C19), its metabolic stability remains uncertain. To overcome this, future research should explore advanced drug delivery systems such as nanoparticle‐based formulations, magnetic nanoparticle, liposomes, or solid lipid nanoparticles, which can enhance solubility, protect the compound from premature degradation, and enable targeted delivery to tumor tissues (Kumari et al. 2023; Zhuo et al. 2024). Additionally, polymeric micelles or phytosome complexes could be considered to improve oral bioavailability and therapeutic index (Barani et al. 2021; Chen et al. 2024; Xu et al. 2013). These strategies would not only improve pharmacokinetic performance but also support controlled release, reduced off‐target toxicity, and improved patient compliance—essential steps in translating Nyctanthic Acid into a clinically viable anticancer agent.

In vitro experiments validated these computational predictions by testing the compounds in cellular models. The results from in vitro experiments demonstrated that the compounds with smaller HOMO‐LUMO gaps from DFT calculations exhibited stronger cytotoxicity, supporting the theory that higher reactivity correlates with enhanced anticancer activity.

## 5 Limitations and Future Prospective

While this study integrates extensive in silico and in vitro methodologies to evaluate the anticancer potential of QI and its key constituents, some limitations remain. First, although molecular dynamics simulations and docking provide insight into stability and binding affinity, they cannot fully recapitulate the complex tumor microenvironment. Second, the in vitro cytotoxicity was assessed only against a single oral cancer cell line (KB), which limits the generalizability of the findings across other subtypes or cancer stages. Additionally, the extract used comprises a mixture of bioactive compounds, and the synergistic or antagonistic effects between these constituents remain unexplored. Without fractionation or compound‐specific validation (e.g., HPLC, GC–MS, and NMR), the precise contribution of individual constituents (Nyctanthic acid and β‐glucogallin) to the observed bioactivity is unclear. However, M. Puppala et al. described the isolation and characterization method of β‐glucogallin in their article (Puppala et al. 2012). Similarly, the extraction, isolation, and characterization process of oleanolic acid (structural and chemical similarity with Nyctanthic acid) was explained by Castellano et al. (Castellano et al. 2022). Essential pharmacokinetic parameters—ADME—as well as systemic toxicity and oral bioavailability were not assessed in vivo. Triterpenoid acids like Nyctanthic acid often face issues with poor aqueous solubility and limited oral bioavailability, which can significantly hamper their clinical efficacy. Additionally, the toxicity profile of Nyctanthic acid has not yet been comprehensively evaluated; although preliminary in vitro assays suggest cytotoxicity toward cancer cells, its effects on normal oral epithelial or systemic cells must be assessed to ensure safety. Further, scalability and standardization of the compound's extraction or synthetic production need to be established for regulatory approval and consistent dosing. The robust preclinical in vivo studies followed by Phase I clinical trials would be essential to evaluate the compound's pharmacodynamics, toxicity thresholds, and therapeutic window. We acknowledge a limitation on DFT results which could not relate to the in vitro cytotoxic activity because we used plant extract for activity. But, DFT results support the molecular docking affinity.

These limitations highlight the need for further experimental validations, including animal studies and clinical trials. To address these limitations and transition toward translational application, future studies should incorporate in vivo validation using appropriate animal models. The 4‐nitroquinoline 1‐oxide (4NQO)‐induced oral cancer model in mice is widely accepted and closely mimics the histopathological and molecular progression of human oral cancer, making it a suitable preclinical model for evaluating QI's efficacy and safety (Kanojia and Vaidya 2006; Tang et al. 2004). We have plans to include additional oral cancer lines (e.g., SCC25, CAL27, cisplatin‐resistant and diverse OC) in future studies to improve generalizability and efficacy.

## 6 Conclusion

This study demonstrated that *Quercus infectoria* possesses promising anticancer potential against oral cancer, with Nyctanthic acid emerging as a key bioactive compound targeting the PI3K‐Akt pathway through stable interaction with AKT1 (−125.426 kcal/mol). The strong in vitro cytotoxicity (IC_50_ value of QI extract is 224.41 ± 2.01 μg/mL) and favorable pharmacokinetic and safety profiles underscore its relevance as a potential lead for drug development. Unlike generic phytotherapeutics, the integrated in silico–in vitro validation provides a mechanistic basis for its anticancer activity. However, to advance toward clinical application, future research must focus on in vivo efficacy studies, pharmacokinetic profiling in animal models, and formulation strategies to address solubility limitations. These steps are essential for transitioning Nyctanthic acid from a promising phytochemical to a clinically viable anticancer candidate.

## Author Contributions

**Priyanka Kamaria:** conceptualization (equal), data curation (lead), formal analysis (equal), methodology (lead), resources (lead), writing – original draft (equal). **Priyanka Tiwari:** formal analysis (supporting), investigation (supporting), resources (supporting), writing – review and editing (supporting). **Prabitha Prabhakaran:** software (supporting), visualization (supporting), writing – review and editing (supporting). **Sakshi Bhardwaj:** software (equal), visualization (equal), writing – review and editing (supporting). **Krishna Kolachi:** software (equal), validation (equal), visualization (equal), writing – review and editing (supporting). **Shankar Thapa:** supervision (equal), visualization (equal), writing – review and editing (lead).

## Ethics Statement

The authors have nothing to report.

## Conflicts of Interest

The authors declare no conflicts of interest.

## Supporting information


**Data S1:** fsn370857‐sup‐0001‐DataS1.docx.

## Data Availability

Available in Supporting Information.
