# Supplementary material for: Evaluation of Quercus infectoria Phytoconstituents Against Oral Cancer: Network Pharmacology, Docking Simulation, and In Vitro Cytotoxicity Assay
Source: Food Sci Nutr. 2025 Sep 8;13(9):e70857. doi: 10.1002/fsn3.70857 (PMC12417334; doi:10.1002/fsn3.70857)
Supplement: Supplementary file 1 — Data S1: fsn370857‐sup‐0001‐DataS1.docx. [file FSN3-13-e70857-s001.docx]

**Research article**

**Evaluation of *Quercus infectoria* Phytoconstituents Against Oral Cancer: Network Pharmacology, Docking Simulation, and In Vitro Cytotoxicity Assay**

Priyanka Kamaria^a^, Priyanka Tiwari^a^, Prabitha Prabhakaran^b^, Sakshi Bhardwaj^c^, Krishna Kolachi^b^, Shankar Thapa^d,e*^

^a^Department of Pharmaceutical Chemistry, KLE College of Pharmacy, Bangalore-560010, KLE Academy of Higher Education and Research, Belagavi-590010, Karnataka, India

^b^Department of Pharmaceutical Chemistry, JSS College of Pharmacy, Mysore Rd, Mysuru, Karnataka-570015

^c^Department of Life Science, Altem Technologies Pvt. Ltd, 5^th^ block Jaya Nagar, Bangalore, Karnataka -560041

^d^Department of Pharmacy, Universal College of Medical Sciences, Bhairahawa, Nepal

^e^Department of Pharmaceutical Chemistry, East Point College of Pharmacy, Bengaluru, India

Corresponding Author: Shankar Thapa, Department of Pharmacy, Universal College of Medical Sciences, Bhairahawa, Nepal

Email id: [tshankar551@gmail.com](mailto:tshankar551@gmail.com)

**Supplementary File**

**Table 1S:** Physicochemical properties of selected phytoconstituents from *Quercus infectoria*. The table presents key drug-likeness parameters, including molecular weight (g/mol), topological polar surface area (TPSA, in Å²), predicted blood–brain barrier (BBB) permeability, number of hydrogen bond donors (HBD), and hydrogen bond acceptors (HBA). These properties were used to evaluate oral bioavailability, membrane permeability, and central nervous system accessibility of each compound during ADMET profiling**.**

| **SN** | **Phytoconstituents** | **Molecular Weight** | **TPSA (Å^2^)** | **BBB** | **HBD** | **HBA** |
| --- | --- | --- | --- | --- | --- | --- |
| 1 | Syringic acid | 198.17 | 75.99 | No | 2 | 4 |
| 2 | Flavylium | 207.25 | 11.3 | Yes | 0 | 0 |
| 3 | β-Glucogallic acid | 332.26 | 177.14 | No | 7 | 9 |
| 4 | Ellagic acid | 302.19 | 141.34 | No | 4 | 8 |
| 5 | Amentoflavone | 538.46 | 181.8 | No | 6 | 10 |
| 6 | Nyctanthic acid | 440.71 | 8.43 | No | 1 | 1 |
| 7 | Methyl oleanolate | 470.74 | 46.53 | No | 1 | 3 |
| 8 | Tannic acid | 1701.21 | 777.98 | - | 25 | 46 |
| 9 | Gallic acid | 170.12 | 97.99 | No | 4 | 4 |
| 10 | Methyl betulate | 470.74 | 46.53 | No | 1 | 3 |
| 11 | β-Sitosterol | 414.72 | 8.02 | No | 1 | 1 |

**
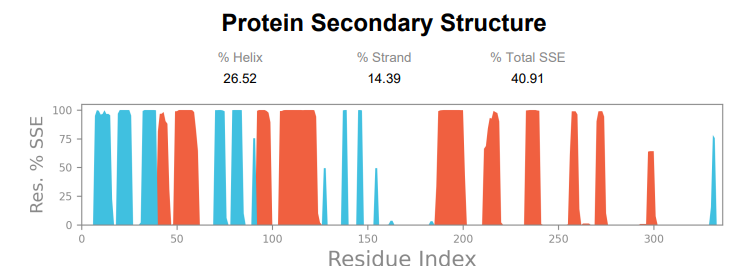
**

**FIGURE 1S** Protein secondary structure distribution of the target protein. The graph illustrates the secondary structure elements along the residue index of the protein. Helices are shown in blue and β-strands in red, representing 26.52% and 14.39% of the protein structure, respectively. The total secondary structure content (SSE) accounts for 40.91% of the entire protein. The y-axis denotes the percentage contribution of each residue to the respective secondary structural element.

**
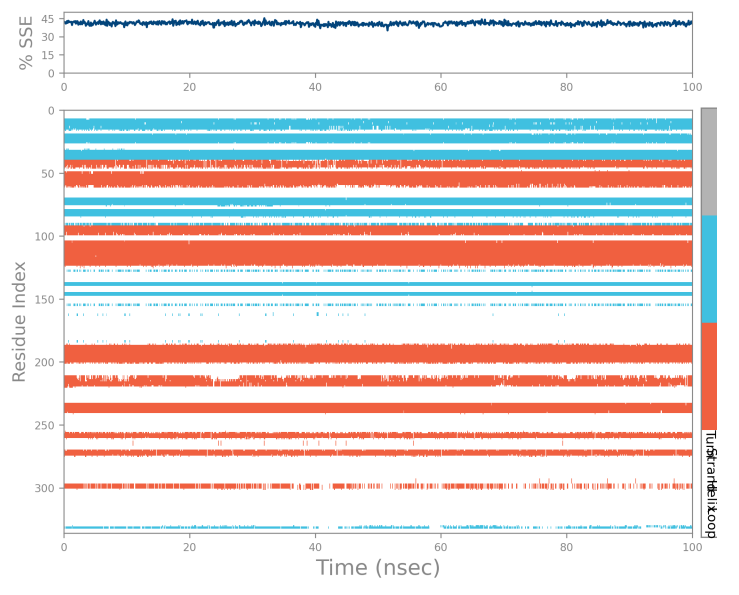
**

**FIGURE 2S** Secondary structural residual plot. Alpha-helices and beta-strands are examples of protein secondary structural elements (SSE) that are tracked during the simulation. The SSE distribution across the protein structure is shown in the plot above by residue index. The SSE composition for every trajectory frame during the simulation is summarized in the figure and each residue's SSE assignment is tracked over time in the plot at the bottom.

**
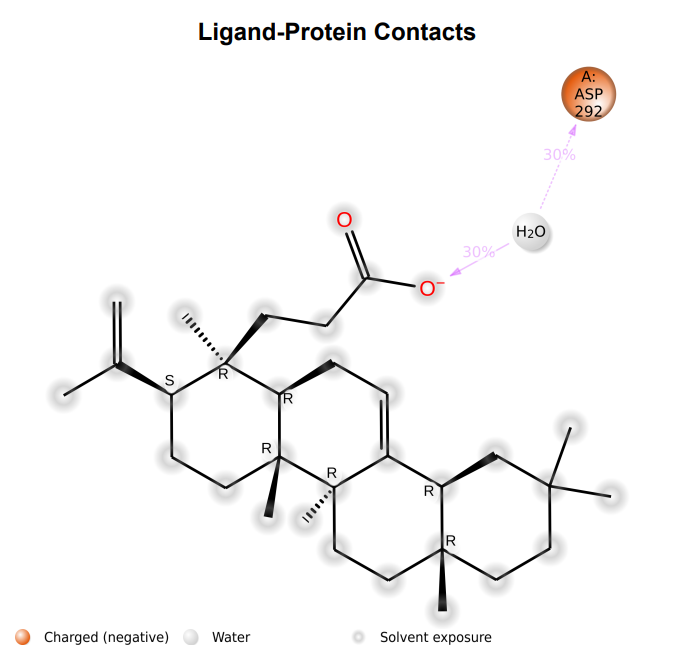
**

**FIGURE 3S** Protein-ligand contact. A schematic FIGURE 3S depicts the detailed ligand atom interactions with the protein residues, which are prominent for a period of 30% of the simulation time and therefore contributes to its biological activity.

**
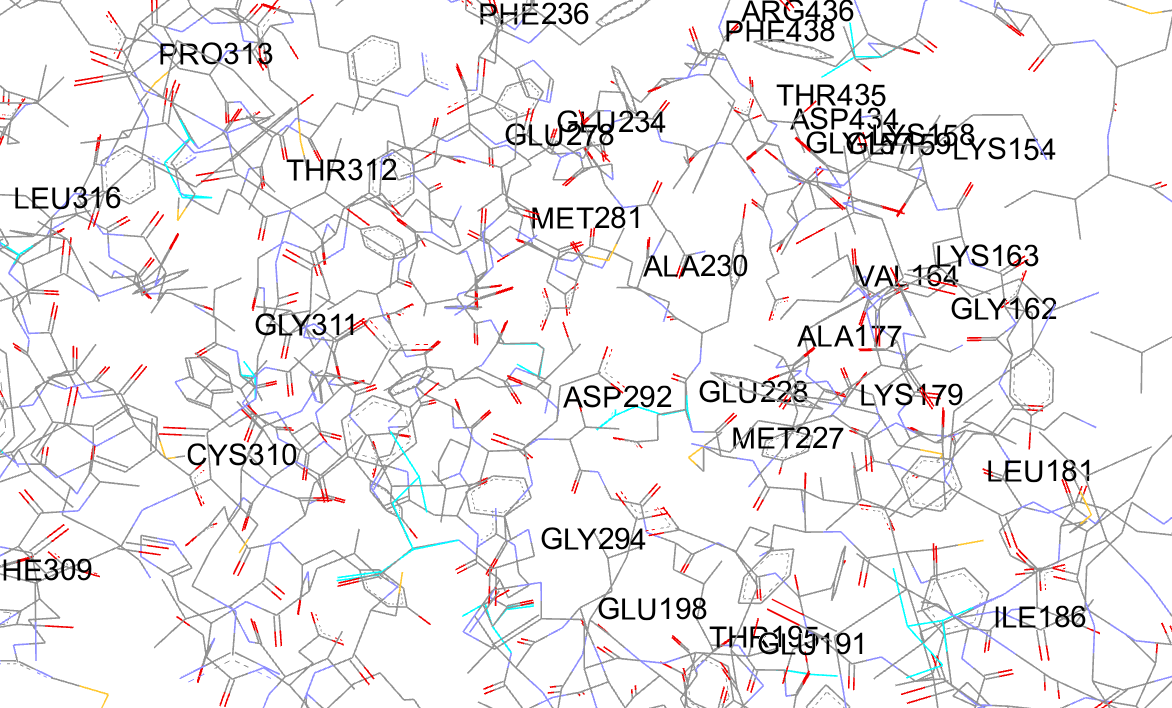
**

**FIGURE 4S** Visualization of binding site residues of AKT1 (PDB ID: 4GV1). The figure highlights the amino acid residues located within the binding pocket of the AKT1 protein, which are potentially involved in ligand interaction and stabilization. Key residues are labeled for clarity, including ASP292, GLU228, MET227, LYS179, ALA177, and surrounding residues such as CYS310, GLY311, and THR312, which may contribute to the structural integrity of the binding site. The image shows the three-dimensional spatial distribution of side chains and backbone atoms within the pocket. Red dashes represent potential hydrogen bond donors or acceptors (such as polar side chains or backbone atoms), while blue and black lines illustrate bond connectivity and structural orientation of residues. This detailed visualization supports the identification of critical contact points between the protein and ligand during docking and simulation studies.
